# Carbomer-based adjuvant elicits CD8 T-cell immunity by inducing a distinct metabolic state in cross-presenting dendritic cells

**DOI:** 10.1371/journal.ppat.1009168

**Published:** 2021-01-14

**Authors:** Woojong Lee, Brock Kingstad-Bakke, Brett Paulson, Autumn Larsen, Katherine Overmyer, Chandranaik B. Marinaik, Kelly Dulli, Randall Toy, Gabriela Vogel, Katherine P. Mueller, Kelsey Tweed, Alex J. Walsh, Jason Russell, Krishanu Saha, Leticia Reyes, Melissa C. Skala, John-Demian Sauer, Dmitry M. Shayakhmetov, Joshua Coon, Krishnendu Roy, M. Suresh

**Affiliations:** 1 Department of Pathobiological Sciences, University of Wisconsin-Madison, Madison, Wisconsin, United States of America; 2 Morgridge Institute for Research, University of Wisconsin-Madison, Madison, Wisconsin, United States of America; 3 Department of Biomolecular Chemistry, University of Wisconsin-Madison, Madison, Wisconsin, United States of America; 4 The Wallace H. Coulter Department of Biomedical Engineering at Georgia Institute of Technology and Emory University and The Parker H. Petit Institute for Bioengineering and Biosciences, Center for ImmunoEngineering, Georgia Institute of Technology, Atlanta, Georgia, United States of America; 5 Department of Biomedical Engineering, University of Wisconsin-Madison, Madison, Wisconsin, United States of America; 6 Wisconsin Institute for Discovery, University of Wisconsin-Madison, Madison, Wisconsin, United States of America; 7 Department of Medical Microbiology and Immunology, University of Wisconsin-Madison, Madison, Wisconsin, United States of America; 8 Lowance Center for Human Immunology, Emory Vaccine Center, Departments of Pediatrics and Medicine, Emory University School of Medicine, Atlanta, Georgia, United States of America; University of Arizona, UNITED STATES

## Abstract

There is a critical need for adjuvants that can safely elicit potent and durable T cell-based immunity to intracellular pathogens. Here, we report that parenteral vaccination with a carbomer-based adjuvant, Adjuplex (ADJ), stimulated robust CD8 T-cell responses to subunit antigens and afforded effective immunity against respiratory challenge with a virus and a systemic intracellular bacterial infection. Studies to understand the metabolic and molecular basis for ADJ’s effect on antigen cross-presentation by dendritic cells (DCs) revealed several unique and distinctive mechanisms. ADJ-stimulated DCs produced IL-1β and IL-18, suggestive of inflammasome activation, but *in vivo* activation of CD8 T cells was unaffected in caspase 1-deficient mice. Cross-presentation induced by TLR agonists requires a critical switch to anabolic metabolism, but ADJ enhanced cross presentation without this metabolic switch in DCs. Instead, ADJ induced in DCs, an unique metabolic state, typified by dampened oxidative phosphorylation and basal levels of glycolysis. In the absence of increased glycolytic flux, ADJ modulated multiple steps in the cytosolic pathway of cross-presentation by enabling accumulation of degraded antigen, reducing endosomal acidity and promoting antigen localization to early endosomes. Further, by increasing ROS production and lipid peroxidation, ADJ promoted antigen escape from endosomes to the cytosol for degradation by proteasomes into peptides for MHC I loading by TAP-dependent pathways. Furthermore, we found that induction of lipid bodies (LBs) and alterations in LB composition mediated by ADJ were also critical for DC cross-presentation. Collectively, our model challenges the prevailing metabolic paradigm by suggesting that DCs can perform effective DC cross-presentation, independent of glycolysis to induce robust T cell-dependent protective immunity to intracellular pathogens. These findings have strong implications in the rational development of safe and effective immune adjuvants to potentiate robust T-cell based immunity.

## Author summary

An adjuvant is the pharmacological agent that is added to vaccines to boost immune responses. Currently, there are only seven FDA-approved adjuvants for human use, and vaccines based on these adjuvants have mainly been evaluated for elicitation of antibody-based immunity. However, vaccines need to also stimulate T cell-mediated immunity to protect against diseases such as AIDS, TB and Malaria. Hence, there is a critical need to develop adjuvants that stimulate protective T cell immunity. Here, we identified an adjuvant (Adjuplex; ADJ) that safely induces strong T cell immunity and protects against virus and intracellular bacteria. We also found that ADJ stimulated T cell immunity by unique mechanisms that did not include metabolic activation of antigen-presenting dendritic cells. Instead, ADJ induced a low metabolic state and engaged mechanisms including lipid pathways and induction of reactive oxygen species to promote activation of T cells by dendritic cells, following vaccination. These data not only provide new mechanistic insights into the mechanisms driving activation of T cells by ADJ, it provides a blue print for what adjuvants need to do to induce protection against infections that require T cell immunity.

## Introduction

Development of effective vaccines remains the central principle for controlling infectious diseases in humans and animals. Typically, vaccines can be classified into the following categories: replicating vaccines (live-attenuated viruses; e.g. smallpox), non-replicating vaccines (subunit [hepatitis B], virus-like particles [human papilloma virus], toxoid [tetanus], and conjugated vaccines [*Haemophilus influenzae* type B]) [1]. To date, protection afforded by the most effective vaccines is primarily dependent upon the elicitation of antibodies [2]. By contrast, development of vaccines against infections that require T cells for pathogen control, such as HIV, tuberculosis (TB), and malaria, remains a difficult challenge for vaccinologists [3–5]. Live-attenuated vaccines are highly immunogenic and elicit both humoral and cell-mediated immunity, but their use can be contraindicated during pregnancy and in immunocompromised individuals [6–8]. Subunit or inactivated antigens are generally safe, but are poorly immunogenic unless formulated in pharmaceutical agents called adjuvants [9].

CD8 T cell responses to non-replicating subunit proteins requires antigen cross-presentation by dendritic cells (DCs) [10]. Likewise, DC cross-presentation plays a pivotal role in eliciting CD8 T cell responses to intracellular pathogens (e.g. *Listeria monocytogenes*) and tumor antigens [11–13]. Cross-presentation of MHC I-restricted antigens to CD8 T cells can occur via vacuolar or cytosolic pathways [14]. In the vacuolar pathway, exogenous antigens are internalized into endosomes and digested by residential cathepsins [15]. By contrast, in the cytosolic pathway, internalized antigens localize to the alkaline endosomal compartment, followed by antigen export into cytosol and downstream processing by the proteasomes [16]. Peptides resulting from proteasomal processing are translocated to endoplasmic reticulum (ER) by the transporter associated with antigen processing-1 (TAP1) complex or to endosomes, and are loaded on to MHC-I molecules [17].

Apart from engaging the appropriate antigen processing cellular machinery, metabolic reprogramming of DCs is an important facet of effective cross-presentation and activation of naïve T cells [18]. DC activation by Toll-like receptor (TLR) agonists triggers a metabolic switch from catabolic metabolism to anabolic metabolism to accommodate increasing cellular demands for executing cellular functions such as production of pro-inflammatory cytokines, upregulation of co-stimulatory molecules, and directed migration to draining lymph nodes [19]. Hence, understanding of DC metabolism is crucial for rationally designed vaccines that can effectively induce robust CD8 T cell responses to subunit protein antigens via mechanisms of DC cross-presentation.

Currently, there are only seven FDA-approved adjuvants for human use, and vaccines based on these adjuvants have mainly been evaluated for elicitation of humoral immunity [20]. There is high level of interest in developing adjuvants that can stimulate potent CD8 and CD4 T-cell responses to subunit antigens. Carbomer (acrylic acid polymers)-based adjuvants (CBA) are components of several veterinary vaccines, and known to safely elicit potent neutralizing antibodies to malarial and HIV envelope glycoproteins in mice and non-human primates [21–23]. We and others have previously reported that the carbomer-based nano-emulsion adjuvant, Adjuplex (ADJ; Advanced Bioadjuvants) protects against influenza A virus in mice [24,25]. However, it is unknown whether ADJ can stimulate protective T-cell immunity to systemic infections, and the mechanisms underlying the stimulation of protective CD8 T cells immunity by ADJ are yet to be determined. Here, we have systematically explored the mechanisms underpinning the molecular and metabolic basis for the potent activation of protective CD8 T cell immunity by an acrylic acid-based nano-emulsion adjuvant, ADJ.

## Results

### ADJ elicits T cell-based protective immunity against viral and intracellular bacterial infections in vivo

**Fig 1:**
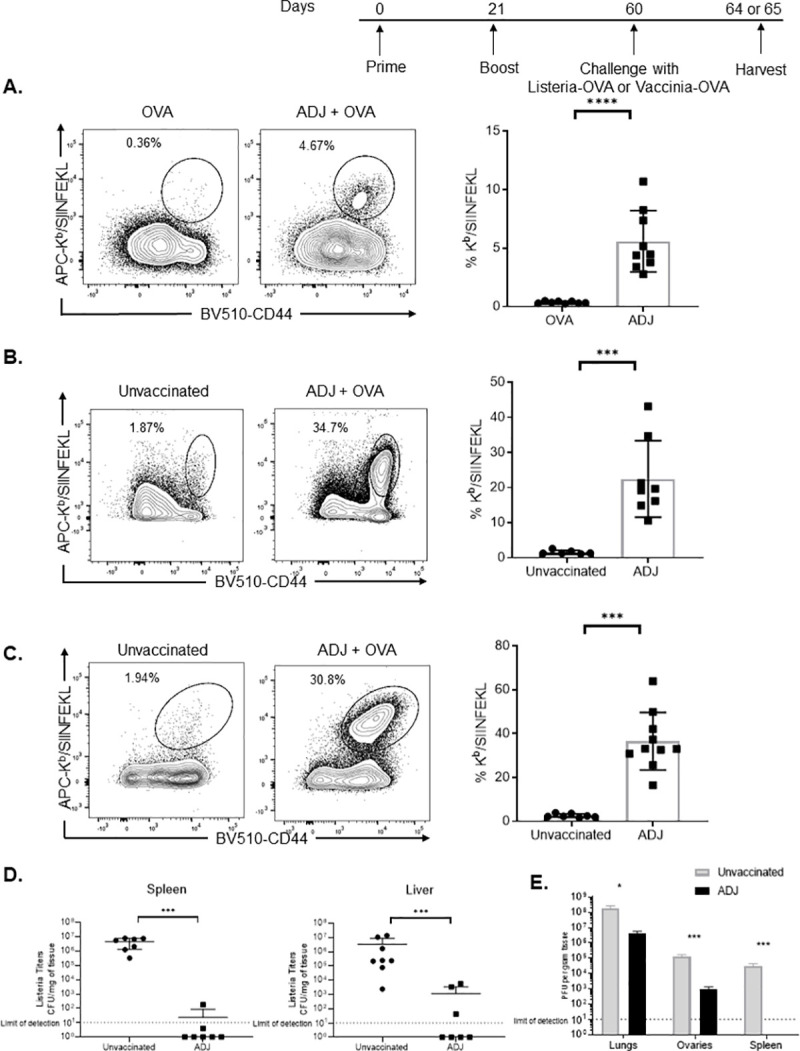
Carbomer adjuvant-based subunit vaccine induces potent CD8 T-cell responses and protects against Listeria and vaccinia challenge *in vivo*. (A) C57BL/6J mice were vaccinated by SQ injection of 10ug OVA ± 5% ADJ formulated in 0.9% sterile saline solution, and boosted 21 days later. At day 8 after last vaccination, activated OVA SIINFEKL-specific CD8 T cells were enumerated in spleens by flow cytometry; FACS plots are gated on live CD8 T cells and show K^b^/SIINFEKL-binding CD44^HI^ CD8 T cells in the gated population. (B-D) Forty days after vaccination, mice were challenged with 1.7x10^5^ CFUs of virulent LM-OVA (Listeria expressing OVA) intravenously or 2x10^6^ PFUs of VV-OVA (Vaccinia Virus-expressing OVA) intranasally; unvaccinated mice (PBS) were challenged as controls. (B) 4 days after LM-OVA challenge, activated OVA SIINFEKL-specific CD8 T cells (FACS plots are gated on CD8 T cells) were quantified in spleen by flow cytometry. (C) 5 days after VV-OVA challenge, activated OVA SIINFEKL-specific CD8 T cells (FACS plots are gated on CD8 T cells) were quantified in lungs by flow cytometry. (D-E) At day 4 (for LM-OVA) or 5 (for VV-OVA) after challenge, LM-OVA burden (spleen and liver) or VV-OVA titers (lungs, ovaries, and spleens) were quantified on BHI agar plates and Vero cells, respectively. Data are pooled from two independent experiments; each data point in bar graphs A, B, C and D represents an individual mouse. Error bars are SEM; *P<0.01; **P<0.001; ***P<0.0001 (Mann-Whitney U test-A-E).

We determined whether ADJ promoted the stimulation of systemic antigen-specific CD8 T-cell responses to an experimental subunit antigen, chicken ovalbumin (OVA). As shown in **Fig 1A**, subcutaneous (SQ) administration of ADJ/OVA formulation potently augmented the activation of OVA-specific CD8^+^ T cells in spleen, while OVA alone was poorly immunogenic. Next, we evaluated whether ADJ-based vaccine conferred T cell-based protection to pathogens, *Listeria monocytogenes* (LM) or vaccinia virus (VV) in mice [26–28]. Forty days after boost, mice were challenged with either recombinant LM-expressing OVA (LM-OVA) or recombinant VV-expressing OVA (VV-OVA) [29,30]. After LM-OVA or VV-OVA challenge, we enumerated recall OVA-specific CD8 T-cell responses in spleens and lungs, and LM-OVA or VV-OVA burden in various tissues. After challenge, higher numbers of OVA SIINFEKL-specific CD8 T cells were detected in spleens or lungs of ADJ+OVA-vaccinated mice, as compared to those in unvaccinated mice **(Fig 1B and 1C)**. Consistent with potent OVA-specific recall CD8 T-cell responses in ADJ+OVA mice, LM-OVA and VV-OVA burden in tissues of ADJ+OVA group were markedly lower than in unvaccinated controls **(Fig 1D and 1E).** In order to benchmark ADJ+OVA-induced protection to LM, we compared the effectiveness of this vaccine approach with previously published live-attenuated LM vaccines [31,32]. In our published work, live-attenuated LM vaccines reduced LM burden by ~4 logs in spleen and liver, as compared to unvaccinated controls [31]. Remarkably, in the current study, LM-OVA burden was below the level of detection in spleen and livers of several ADJ/OVA-vaccinated mice compared to ~10^7^ CFU of LM-OVA in organs of unvaccinated controls (**Fig 1D**). Thus, ADJ-based subunit protein vaccine provided effective systemic protection against Listeria that may be superior to live-attenuated LM vaccines. Together, data in **Fig 1** demonstrated that ADJ-based subunit vaccine provided T-cell-based protective immunity against bacterial and viral pathogens, presumably by promoting cross-presentation of antigen to OVA-specific CD8 T cells *in vivo*.

### Carbomer-based nano-emulsion adjuvant ADJ enhances cross-presentation of antigens by DCs *in vitro* and *in vivo*

**Fig 2:**
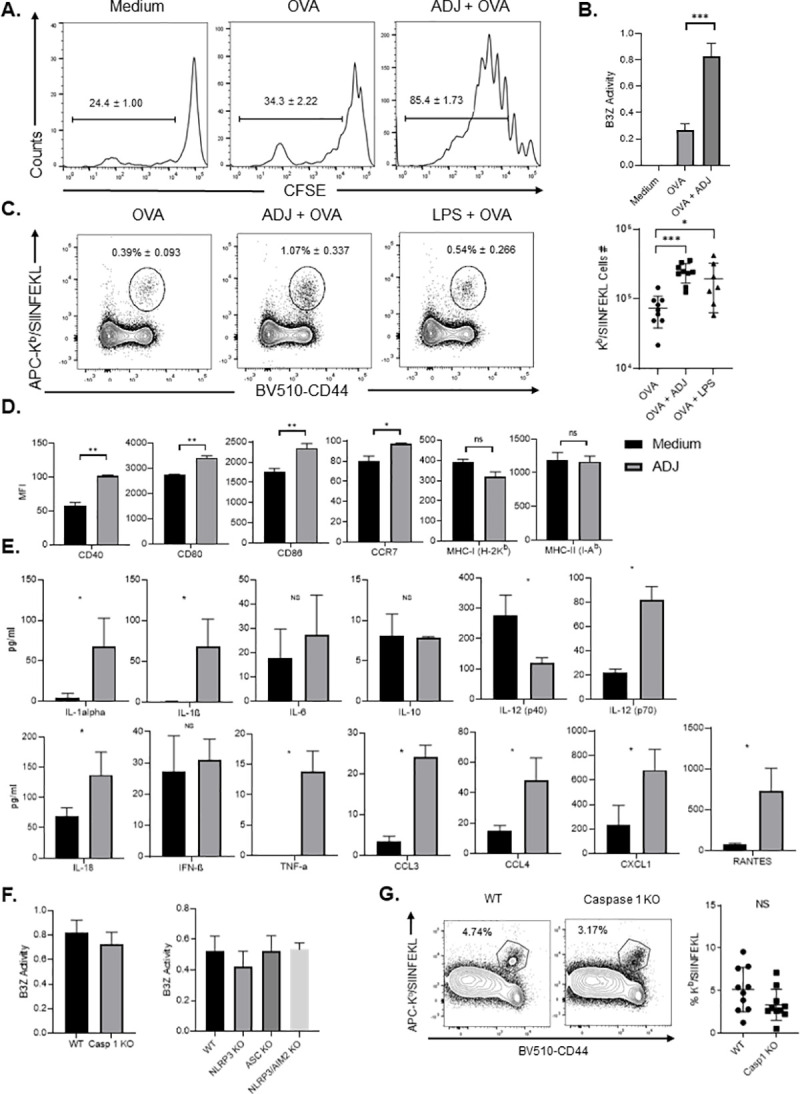
Carbomer-based adjuvants enhance DC cross-presentation, which is independent of co-stimulatory molecules and inflammasome activation. (A) BMDCs (1x10^5^ cells/well) were exposed to media or OVA ± ADJ for 5 h, and co-cultured with CFSE-labeled OT-I CD8 T cells (2x10^5^ cells/well) for 72 h. Histograms are gated on live OT-I CD8 T cells; the numbers are the percentages of gated cells that have reduced levels of CFSE fluorescence. (B) BMDCs (1x10^5^ cells/well) were exposed to media or OVA ± ADJ for 5 h, and co-cultured with B3Z cells (1x10^5^ cells/well) for 24 h. β-galactosidase activity in B3Z cells was quantified by CPRG colorimetry. (C) BMDCs were cultured with OVA ± ADJ or LPS for 6 h, washed three times, and injected *i*.*v*. into C57BL/6 mice. After 7 days, OVA SIINFEKL-specific CD8 T cells were quantified in spleen using K^b^/SIINFEKL tetramers. (D) FACS analysis of CD40, CD80, CD86, CCR7, MHC-I and MHC-II expression in BMDCs after treatment with ADJ for 6 h. (E) Measurement of cytokines in culture media supernatant of BMDCs at 24 hours after stimulation with ADJ, by Multiplex Luminex Assay or ELISA. (F) β-galactosidase production by B3Z cells after co-culture with WT DCs or DCs deficient for AIM2, ASC, NLRP3 or Caspase 1, pre-treated with OVA ± ADJ for 5 h. (G) Wild type and caspase 1 KO mice were vaccinated SQ with OVA (10ug) + ADJ (5%). On the 8th day after vaccination, the percentages of activated OVA SIINFEKL-specific CD8 T cells in the spleen were quantified by flow cytometry. Data in A, D and E are representative of five independent experiments. Data in panels B and G are pooled from two independent in vivo experiments; each data point represents an individual mouse. Error bars show SEM; *P<0.01 (ANOVA test: A-C; Student’s t-test -D-F; Mann-Whitney U test-G).

Next, in order to determine the effects of ADJ on antigen-presenting cells, we assessed whether exposure of bone marrow-derived DCs (BMDCs) to ADJ lead to enhanced antigen cross-presentation to CD8 T cells *in vitro* and *in vivo*. We evaluated the ability of ADJ-treated DCs to activate naïve CD8 T cells to undergo antigen-driven proliferation by culturing BMDCs with either OVA or ADJ+OVA. Subsequently, BMDCs were co-cultured with carboxyfluorescein succimidyl ester (CFSE)-labeled naïve OVA SIINFEKL-specific TCR transgenic OT-I CD8 T cells for 72 hours. BMDCs cultured with media or OVA induced proliferation of a small fraction of OT-I CD8 T cells **(Fig 2A)**. By contrast, BMDCs cultured with ADJ+OVA stimulated proliferation of >80% of OT-I CD8 T cells *in vitro*, as indicated by reduced levels of CFSE fluorescence. To qualitatively assess the magnitude of ADJ-mediated cross presentation of OVA antigen to CD8 T cells *in vitro*, BMDCs were treated with ADJ+OVA or OVA, and then were evaluated for their capacity to activate SIINFEKL-specific B3Z T cell hybridoma cells using a reporter assay [33]. BMDCs stimulated with ADJ+OVA significantly induced β-galactosidase in B3Z cells compared to OVA only control, suggesting enhanced antigen cross-presentation by ADJ-treated BMDCs **(Fig 2B)**. To further corroborate if ADJ promotes cross-presentation by enhancing the expression levels of SIINFEKL/H-2K^b^ complexes on the surface of antigen-presenting cells, we treated BMDCs with media, OVA, or ADJ+OVA, and quantified cell surface SIINFEKL/H-2K^b^ complexes using the 25 D1.16 antibody. Significantly greater percentages of DCs exposed to ADJ+OVA expressed elevated levels of SIINFEKL-bearing H-2K^b^ molecules, in comparison to DCs exposed to media or OVA only **(S1 Fig)**. Next, to assess whether DCs treated with ADJ possess enhanced cross-priming abilities *in vivo*, we adoptively transferred DCs pre-treated *in vitro* with ADJ+OVA, LPS+OVA, or OVA into C57BL/6 mice. The percentages and total numbers of SIINFEKL-specific CD8 T cells were significantly higher in spleens of mice that received DCs treated with ADJ+OVA, as compared to mice that received DCs treated with LPS+OVA or OVA **(Fig 2C).** In summary, data in **Figs 2A, 2B, 2C and S1** strongly suggest that ADJ enhances DC cross-presentation of antigens to CD8 T cells *in vitro* and *in vivo*.

### ADJ induces IL-1β and IL-18 production in DCs, but deficiency for NLRP3, ASC or caspase 1 did not affect ADJ-mediated cross-presentation

Next, we examined the effects of ADJ on the expression profiles of cytokines, chemokines and canonical cell surface markers of BMDC activation. Compared to resting DCs, ADJ-treated DCs showed statistically significant, yet modest increases in expression of CD40, CD80, CD86, and CCR7; no significant differences in expression were observed for MHC-I and MHC–II **(Fig 2D)**. ADJ-treated DCs produced higher levels of IL-12 (p70), TNF-α, IL-1α, CCL3, CCL4, CXCL1, and RANTES, as compared to untreated DCs; no significant differences in expression were observed for IL-6, IL-10, and IFN-β **(Fig 2E)**. Significantly, ADJ-stimulated DCs also produced elevated levels of IL-1β and IL-18 **(Fig 2E)**, which suggests that ADJ treatment leads to inflammasome activation. Since inflammasome activation has been implicated in modulation of antigen presentation by DCs [34,35], we performed B3Z assays using DCs deficient in NLRP3, ASC, or caspase 1 to interrogate whether inflammasome activation is required for ADJ-induced cross-presentation in BMDCs. Loss of NLRP3, ASC or caspase 1 activity did not affect ADJ-induced cross-presentation by DCs, *in vitro* **(Fig 2F)**. To validate whether caspase 1 is required for ADJ-aided cross-presentation *in vivo*, we immunized cohorts of wild type (WT) and caspase 1-deficient (Caspase 1 KO) mice with ADJ+OVA, and quantified OVA SIINFEKL-specific CD8 T cells in spleens using MHC I tetramers at day 8 after immunization. Caspase 1 deficiency did not significantly affect the accumulation of activated SIINFEKL-specific CD8 T cells in spleens **(Fig 2G)**, suggesting that caspase 1 is not essential for ADJ-driven cross-presentation to CD8 T cells *in vivo*.

### ADJ modulates antigen processing and subcellular localization in DCs

**Fig 3:**
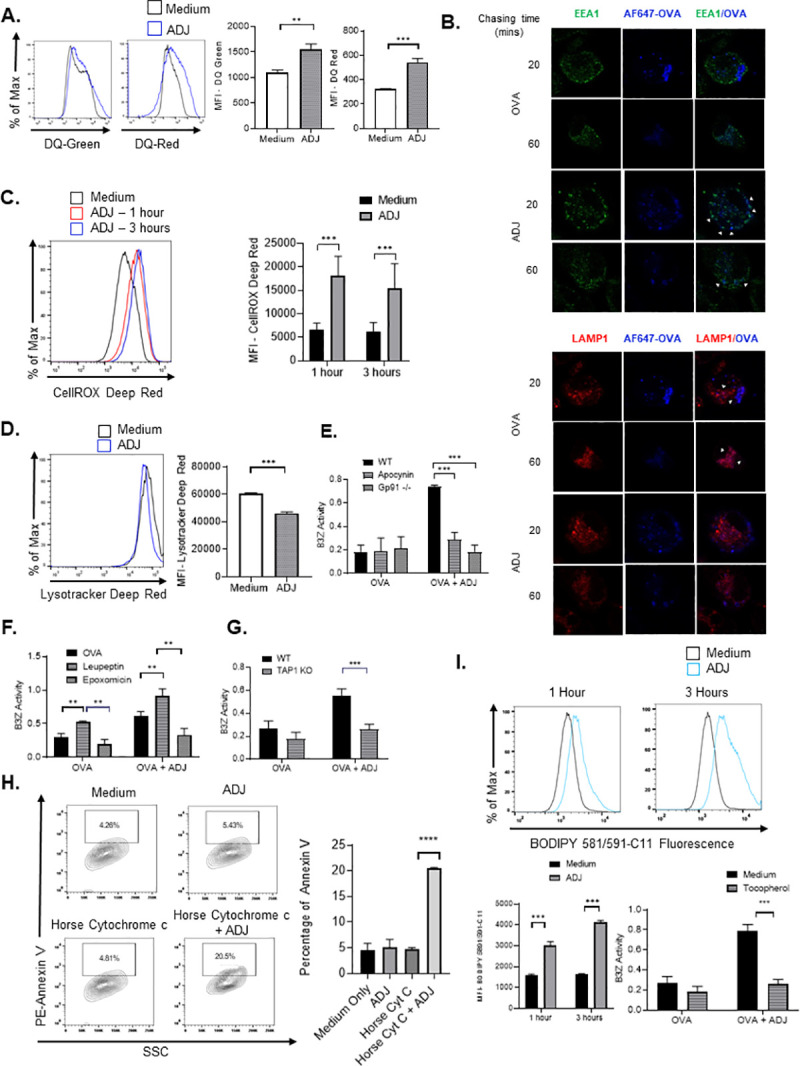
Carbomer-based adjuvant engages the endosome-to-cytosol pathway of cross-presentation. (A) BMDCs (2x10^5^ cells/well) were cultured in media containing DQ-OVA (20ug) ± ADJ (1%) for 6 h and the fluorescence of DQ-green (FITC channel) or DQ-red (PE-Texas Red channel) was quantified using flow cytometry (B) BMDCs were pulsed with Alexa 647-OVA (20ug) ± ADJ (1%), chased at the indicated time-points, and immunostained to assess the degree of OVA co-localization with either EEA1^+ve^ (early endosomes) or LAMP1^+ve^ (lysosomes) organelles. Scale bars, 10 μm. (C-D) BMDCs were cultured in media ± ADJ (1%) for the indicated time (1 or 3 hrs) and stained with CellRox and Lysotracker dyes to measure total cellular ROS and intracellular acidity, respectively. The fluorescence of CellROX and Lysotracker was quantified by flow cytometry. (E) B3Z cross-presentation assay was performed using gp91-deficient (gp91^-/-^) BMDCs or DCs treated ± the NADPH oxidase inhibitor (apocynin). (F, I) β-galactosidase production by B3Z cells after co-culture with BMDCs pre-treated with OVA ± ADJ in the presence/absence of tocopherol (IμM, 100μM), leupeptin (F; 50μM), or epoxomycin (F; 10μM) for 5 h. (G) β-galactosidase production by B3Z cells after co-culture with ADJ/OVA-treated WT or TAP1-deficient DCs pre-treated with OVA ± ADJ for 5 h. (H) ADJ-treated DCs were cultured with horse cytochrome C (5mg/ml) ± ADJ (1%) for 24 hours to assess endosomal leakage of antigen into cytosol. Cell viability (as a read-out for antigen leakage-induced apoptosis) was measured by Annexin V staining. (I) BMDCs were cultured in media ± ADJ (1%) for 1 and 3 h, stained with BODIPY 581/591-C11, and a blue-shift of BODIPY 581/591-C11 fluorescence was measured using flow cytometry. Data are representative of 3 independent experiments. Error bars are the SEM; **P<0.001; ***P<0.0001 (Student’s t-test- A, C, D, G and I; ANOVA test–B, E, F, G, H).

We next examined whether ADJ affected the dynamics of antigen processing in DCs. First, we determined the effect of ADJ on antigen uptake by culturing DCs with OVA that was labeled with pH-insensitive dye, Alexa Fluor 647. Interestingly, we found that antigen uptake was significantly reduced in DCs treated with ADJ **(S2 Fig)**. This finding was not totally unexpected, as some TLR agonists are known to reduce uptake of soluble antigen, yet increase antigen cross-presentation [36]. Next, we assessed whether ADJ affected antigen processing by treating DCs with DQ-OVA, which emits green fluorescence upon proteolytic degradation and red fluorescence upon subsequent aggregation of digested peptides. We found that ADJ enhanced OVA degradation and/or accumulation of processed OVA, as indicated by an increase of both DQ-green and DQ-red fluorescence at 6 hours **(Fig 3A)**. Hence, ADJ might dampen antigen uptake, but enhances antigen processing and/or accumulation of processed antigen in DCs.

Apart from antigen degradation, trafficking of antigens to less acidic vesicular compartments, such as early endosomes, is critical for efficient cross-presentation [37,38]. Hence, we analyzed the effect of ADJ on the intracellular routing of antigen using pHAB-OVA, which emits green fluorescence under acidic conditions. In ADJ-treated DCs, pHAB-OVA was routed to less acidic compartments in DCs within 30 minutes, as indicated by lower fluorescence of pHAB-OVA compared to media control **(S2 Fig)**. In order to precisely localize antigen in ADJ-treated DCs, we investigated the extent to which OVA antigen was localized to early endosomes or lysosomes, using confocal microscopy. Microscopic images showed that in ADJ-treated DCs, antigen preferentially co-localized with early endosomes (EEA1^+ve^), rather than lysosomes (LAMP1^+ve^) **(Fig 3B)**. Unlike in ADJ-treated DCs, antigen was localized to the acidic lysosomes in DCs that were only treated with OVA **(Fig 3B)**. To further corroborate our findings, we employed a co-localization analysis of confocal microscopy images using Pearson's correlation coefficients (PCC) [39]. This analysis showed that the PCC of EEA1 with OVA was higher, but the PCC of LAMP1 with OVA was lower in ADJ-treated DCs **(S3 Fig).** Results indicated that a higher proportion of EEA1^+ve^ organelles but not LAMP1^+ve^ organelles co-localized with antigen in ADJ-treated cells. Collectively, these findings suggested that ADJ preferentially promotes antigen localization to less acidic early endosomal compartments.

### ADJ enhances antigen cross-presentation by inducing ROS production and modulating the pH of the antigen-containing compartment by NOX2-dependent mechanisms

Augmented ROS production reduces endosomal acidity and delays antigen degradation, which in turn enhances antigen cross presentation [40]. Here, we used CellROX reagent and the ROS-Glo H_2_O_2_ assay to quantify endosomal ROS and H_2_O_2_ production, respectively in ADJ-treated BMDCs. Remarkably, cellular ROS and H_2_O_2_ levels rapidly increased within 1 hour after treatment with ADJ (**Figs 3C and S4).** Next, we asked whether alterations in cellular ROS modulated intracellular acidity in ADJ-treated DCs; Lysotracker has been used for visualizing acidic compartments. Within 1 hour of ADJ treatment, concomitant to ROS induction, there was a substantive reduction of Lysotracker MFI, indicating that ADJ might have induced ROS and reduced intracellular acidity in DCs **(Fig 3D).** In order to test the importance of ADJ-induced ROS in cross-presentation, we employed a combination of pharmacological and genetic approaches. First, pharmacological inhibition of NADPH-oxidase complex2 (NOX2) assembly with apocynin markedly reduced DC cross-presentation to B3Z cells **(Fig 3E).** Furthermore, unlike strong B3Z activation in ADJ-treated WT DCs, ADJ failed to augment cross-presentation in DCs deficient for the ROS-inducing NOX2 complex component gp91 **(Fig 3E).** Data in **Fig 3C, 3D and 3E** support the idea that maintenance of a less acidic environment in the endosomes established by NOX2-driven ROS might be critical for ADJ-mediated cross-presentation.

### ADJ-induced cross-presentation requires proteasomal processing and TAP1 transporters

MHC-I binding peptides can be generated either by phagosomal residential cathepsins, or by cytosolic proteasomes. In order to dissect the pathways required for cross-presentation of OVA-derived peptides by ADJ-treated DCs, we inhibited proteasomal or lysosomal activities using epoxomycin (proteasomal inhibitor) or leupeptin (general cathepsin inhibitor), respectively. ADJ-driven cross-presentation was effectively abrogated by epoxomycin, but augmented by leupeptin, as compared to vehicle controls **(Fig 3F).** These findings suggested that ADJ-driven antigen cross-presentation required proteasomal, but not lysosomal activity. Next, we investigated whether ADJ enhanced proteasomal activity in DCs using Cell-Based Proteasome-Glo Assays. Here, we found that the activity of 20S proteasome subunits were not affected by ADJ treatment **(S5 Fig).** Thus, ADJ-mediated cross-presentation requires cytosolic proteasomes, but ADJ does not enhance the proteolytic activity of proteasomes in DCs.

Peptides generated by cytosolic proteasomes require TAP transporters to access MHC I molecules in ER or ER-Golgi intermediate compartment (ERGIC) [41,42]. We tested the extent to which ADJ-induced DC cross-presentation is dependent upon TAP transporters. ADJ-mediated DC cross-presentation of OVA peptides and antigen recognition by B3Z cells was completely abolished in the absence of TAP1 (**Fig 3G**), which suggested that ADJ-driven DC cross-presentation required TAP1 for loading peptides on to MHC I. Collectively, our data suggest that ADJ induction of DC cross-presentation requires an endosomes-to-cytosol pathway of cross-presentation.

### ADJ-mediated cross-presentation requires endosomal antigen leakage mediated by lipid peroxidation

The cytosolic pathway of cross-presentation requires antigen escape from endosome into cytosol, for subsequent degradation by cytosolic proteasomes [16]. Because we observed that ADJ-induced DC cross-presentation required proteasomes as antigen processing machinery **(Fig 3F),** we questioned whether ADJ promoted antigen translocation from endosomes into cytosol. Endosomal leakage in DCs was visualized by measuring cellular apoptosis, resulting from release of exogenous horse cytochrome C (cytc) into the cytosol [43]. After 24 hours of treatment with cytc in the presence or absence of ADJ, we found that the percentages of annexin-V positive cells were 4 times higher among DCs exposed to ADJ+cytc, compared to cytc- or ADJ-treated DCs. These data suggested that ADJ likely induced cytc escape from endosomes into cytosol, resulting in DC apoptosis **(Fig 3H).**

It was recently reported that NOX2-driven ROS can cause endosomal lipid peroxidation and release of antigen from leaky endosomes into the cytosol of DCs [44]. To determine if observed endosomal leakage in ADJ-treated DCs **(Fig 3H)**, was caused by lipid peroxidation, we quantified lipid peroxidation in ADJ-treated DCs using a radiometric dye, BODIPY 581/591 C11, which displays a shift in peak fluorescence emission from red to green upon oxidation by lipid hydroperoxides. Within 1 hour after ADJ treatment, there was a marked shift of BODIPY 581/591 C11 from red to green **(Fig 3I).** Moreover, pharmacological inhibition of lipid peroxidation using α-tocopherol, a lipid-soluble antioxidant which selectively prevents lipid peroxidation by scavenging free electrons [45], significantly reduced ADJ-driven cross-presentation and activation of B3Z cells **(Fig 3I).** Thus, our data illustrated that ADJ-mediated cross-presentation required ROS, and likely lipid peroxidation for facilitating endosomal antigen leakage.

### ADJ enhances cross-presentation without glycolytic reprogramming of DCs via the Akt-mTORC1-KLF2-HIF-1α axis

Typically, catabolic metabolism in resting DCs is characterized by oxidative phosphorylation (OXPHOS) fueled by fatty acid oxidation (FAO) and limited glycolysis [46–48]. During early DC activation by TLR agonists, DCs augment both aerobic glycolysis and OXPHOS to support the anabolic demands required for expansion of the ER and Golgi apparatus, *de novo* fatty acid (FA) synthesis, and production of inflammatory cytokines. Further, this early glycolytic reprogramming by TLRs is required for upregulation of co-stimulatory molecules, CCR7 oligomerization, and priming T cells [49,50]. Subsequently, DCs inhibit OXPHOS via nitric oxide (NO) and rely on aerobic glycolysis for their survival, especially after sustained exposure to TLR-agonists [51,52]. Based on the augmented ability of ADJ-treated DCs to activate T cells, we hypothesized that metabolic reprogramming plays a distinctive role in this process.

**Fig 4:**
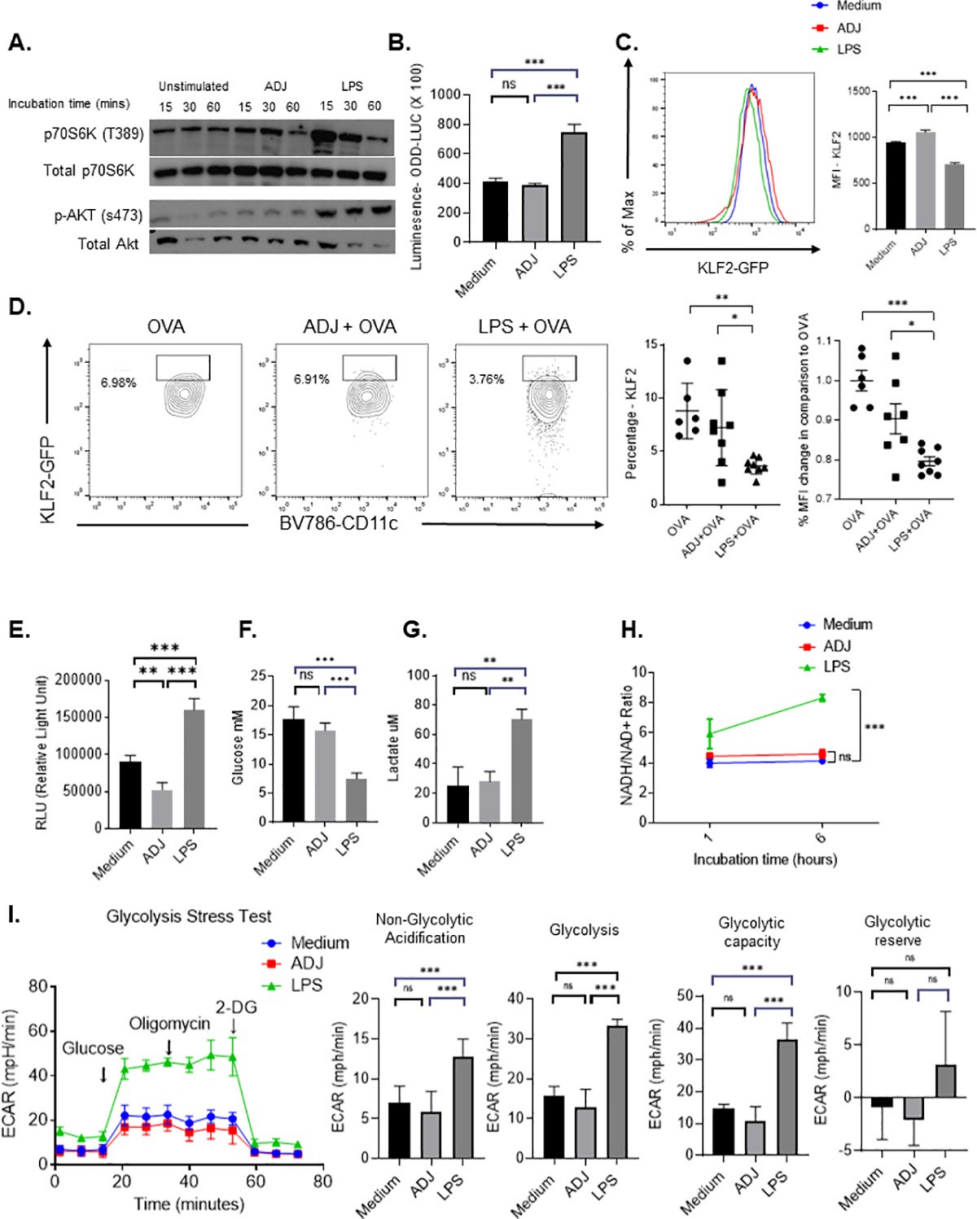
Carbomer-based adjuvant rewires DC metabolism without engaging aerobic glycolysis driven by the Akt-mTORC1-KLF2-HIF1α signaling axis. (A) Immunoblot for total AKT and AKT phosphorylated at Ser473 or total p-S6K and p-S6K phosphorylated at Thr389 in BMDCs following 15, 30, 60 mins of culture with and without ADJ (1%) or LPS (100ng/ml). (B) HIF-1α induction was measured by quantifying luciferase activity in DCs derived from ODD-Luc mice; DCs were stimulated with ADJ (1%) or LPS (100ng/ml) for 24 h. (C) *In vitro* KLF2 expression in DCs; DCs derived from KLF2-GFP mice were treated with or without ADJ (1%) or LPS (100ng/ml) for 24 hours, and KLF2-GFP expression was assessed by flow cytometry (D) KLF2-GFP reporter expression (gated on CD11c^HI^/MHC-II^HI^ cDC population) was measured in DCs from inguinal lymph nodes after 24 h of intradermal footpad vaccination. (E) Intracellular ATP was quantified in unstimulated, ADJ- or LPS-stimulated DCs at 24 h. (F-G) Extracellular glucose levels and secreted lactate in cell culture supernatant were measured in cultures of unstimulated, ADJ-, and LPS-stimulated DCs at 24 h. (H) NADH/NAD+ ratio in resting, ADJ- or LPS-stimulated DCs at the indicated time-points (1 and 6 hours). (I) BMDCs were treated with ADJ (1%) or LPS (100ng/ml) for 24 h; real-time ECAR was determined during sequential treatments with glucose, oligomycin, and 2-DG. Quantification of basal glycolysis (ECAR), glycolytic capacity and glycolytic reserve. The glycolytic reserve capacity of cells was measured as the difference between ECAR before and after addition of oligomycin. Data are representative of 2–3 independent experiments (A, B, C, E-I). Data in D are pooled from two independent in vivo experiments and each symbol represents an individual mouse. Error bars show SEM; *P<0.01; **P<0.001; ***P<0.0001 (B-I; one-way ANOVA).

First, we asked whether ADJ engaged aerobic glycolysis as a key source of carbon for metabolic functions that enhance antigen presentation by DCs via the Akt-mTORC1-HIF-1α signaling axis. LPS stimulation potently triggered phosphorylation of p70S6K and Akt within 60 minutes, but treatment with ADJ failed to do the same **(Fig 4A)**. Prolonged glycolytic reprogramming involves the induction and stabilization of hypoxia-inducible factor (HIF-1α), that in turn trigger production of NO and suppression of OXPHOS [53,54]. Using DCs from HIF-1α luciferase reporter mice [55], we compared ADJ and LPS for HIF-1α induction. Only stimulation with LPS, but not ADJ, induced HIF-1α in DCs **(Fig 4B)**. Kruppel-like factor 2 (KLF2) is a transcription factor that inhibits the expression and transcriptional activity of HIF-1α, and downregulation of KLF2 is associated with engagement of glycolysis in immune cells [56]. As another measure of glycolytic reprogramming, we assessed KLF2 expression levels using BMDCs from KLF2-GFP reporter mice [57]. High levels of KLF2 were detected in unstimulated DCs, and KLF2 expression was significantly downregulated in LPS-stimulated DCs, but not in ADJ-treated DCs **(Fig 4C).** To confirm these findings *in vivo*, we immunized KLF2-GFP mice with OVA, ADJ+OVA or LPS+OVA and examined DC KLF2 expression in vaccine-draining lymph nodes. As shown in **Fig 4D**, DCs from mice immunized with LPS+OVA, but not ADJ+OVA, showed significant down-regulation of KLF2 expression, as compared to DCs from OVA only mice. Thus, unlike LPS, ADJ failed to engage the Akt-mTORC1-KLF2-HIF-1α signaling pathway in DCs.

To directly investigate the effect of ADJ on DCs’ glycolytic metabolism, we quantified intracellular ATP levels in DCs treated with ADJ or LPS. LPS-treated DCs, but not ADJ-treated DCs contained higher levels of ATP than untreated DCs **(Fig 4E).** As an index of glycolysis in ADJ- and LPS-treated DCs, we quantified glucose consumption and lactate production *in vitro*. The extracellular concentrations of glucose and lactate were unaffected by ADJ stimulation, suggesting that ADJ did not alter glucose utilization or lactate production by DCs **(Fig 4F and 4G).** Further, we did not find significant alteration of the NADH/NAD+ ratio in ADJ-treated DCs, which suggested that ADJ exposure did not cause a cellular redox imbalance **(Fig 4H)**. Lastly, we quantified the functional glycolytic capacity of ADJ-stimulated DCs using the glycolysis stress test. We found that neither the glycolytic capacity nor the glycolytic reserves were altered in ADJ-treated DCs, as compared to those in unstimulated DCs, while LPS up-regulated both glycolytic capacities and reserves in DCs **(Fig 4I).** Together, data in **Fig 4** strongly suggested that ADJ-mediated cross-presentation occurred independent of enhanced aerobic glycolysis.

### ADJ disengages OXPHOS in DCs by iNOS-independent mechanisms

**Fig 5:**
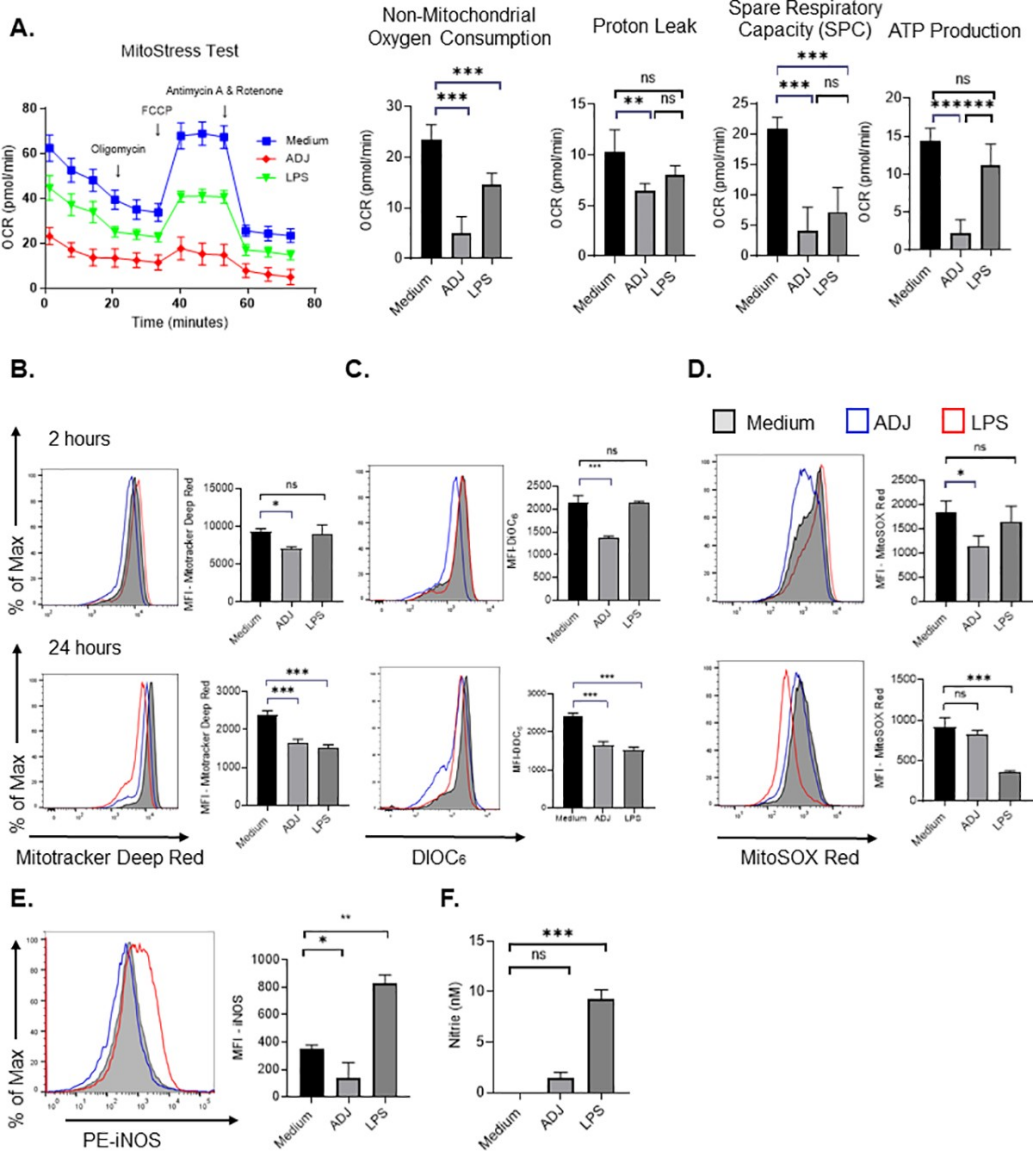
Carbomer-based adjuvant suppresses oxidative phosphorylation in DCs by iNOS-independent mechanisms. (A) BMDCs (7x10^4^ cells/well) were treated with media, ADJ (1%) or LPS (100ng/ml) for 24 h; real-time OCR was determined during sequential treatments with oligomycin, FCCP, and antimycin-A/rotenone. Basal respiration, maximal respiration and spare respiratory capacity (SRC). SRC were calculated as the difference in OCR after addition of FCCP and OCR before the addition of oligomycin. (B-D) BMDCs (2x10^5^ cells/well) were treated with media, ADJ (1%) or LPS (100ng/ml) for the indicated time periods (2 or 24 h) and stained with Mitotracker Deep Red, DiOC_6_, MitoSOX Red Mitochondrial Superoxide Indicator to quantify mitochondrial mass, membrane potential or mitochondrial superoxide, respectively. (E) Culture supernatants from DCs cultured with ADJ (1%) or LPS (100ng/ml) were analyzed for nitrite using Griess reagents. (F) iNOS expression in unstimulated, ADJ (1%)- or LPS (100ng/ml)-stimulated DCs at 24 h, was determined by intracellular staining using Cytofix/perm kit. Data are representative of 2–3 independent experiments. Error bars are SEM; *P<0.01; **P<0.001; ***P<0.0001 (A-F; one-way ANOVA).

Because ADJ failed to augment glycolysis **(Fig 4)**, we interrogated whether ADJ engaged OXPHOS as an alternative metabolic pathway. By performing extracellular flux analysis, we measured alterations in oxygen consumption in real time **(Fig 5A)**. At 24 hours after stimulation, the mitochondrial oxygen consumption rate (OCR) was highest in unstimulated DCs [58] and mitochondrial OCR was lower in LPS-treated DCs. Notably, even baseline OCR for ADJ-treated DCs was markedly lower than in unstimulated DCs, and failed to show detectable increase, following addition of FCCP, a potent mitochondrial un-coupler that disrupts ATP synthesis **(Fig 5A)**. To further evaluate ADJ’s effects on mitochondrial metabolism, we measured mitochondrial content, membrane potential (Ψm) and mitochondrial superoxide production (mROS). Within 2 hours of ADJ treatment, we observed a drastic reduction in mitochondrial content, Ψm, and mROS, in comparison to both resting and LPS-stimulated DCs **(Fig 5B, 5C and 5D)**. Interestingly, loss of mitochondrial functions persisted over 24 hours in ADJ-treated cells, indicating that ADJ suppressed mitochondrial functions at both early and late stages of stimulation. These data are consistent with a decrease in spare respiratory capacity **(Fig 5A)** in ADJ-stimulated DCs, and support the inference that ADJ-mediated metabolic programming includes a profound decline in mitochondrial activity.

One of the mechanisms for inhibiting mitochondrial functions in DCs is the induction of NO, which interferes with electron transport chain by blocking oxygen consumption and ATP production [59]. We probed whether impairment of mitochondrial function by ADJ was linked to NO induction in DCs. The cellular levels of inducible nitric oxide (iNOS) **(Fig 5E)** and extracellular nitrite levels **(Fig 5F)** in the supernatant of ADJ-treated DCs did not vary, in comparison to unstimulated DCs, while LPS-stimulated DCs contained elevated levels of cellular iNOS and extracellular nitrite. In summary, these data collectively suggested that ADJ disengaged mitochondrial functions by mechanisms independent of iNOS.

### ADJ-induced intracellular lipid body formation is required for DC cross-presentation

**Fig 6:**
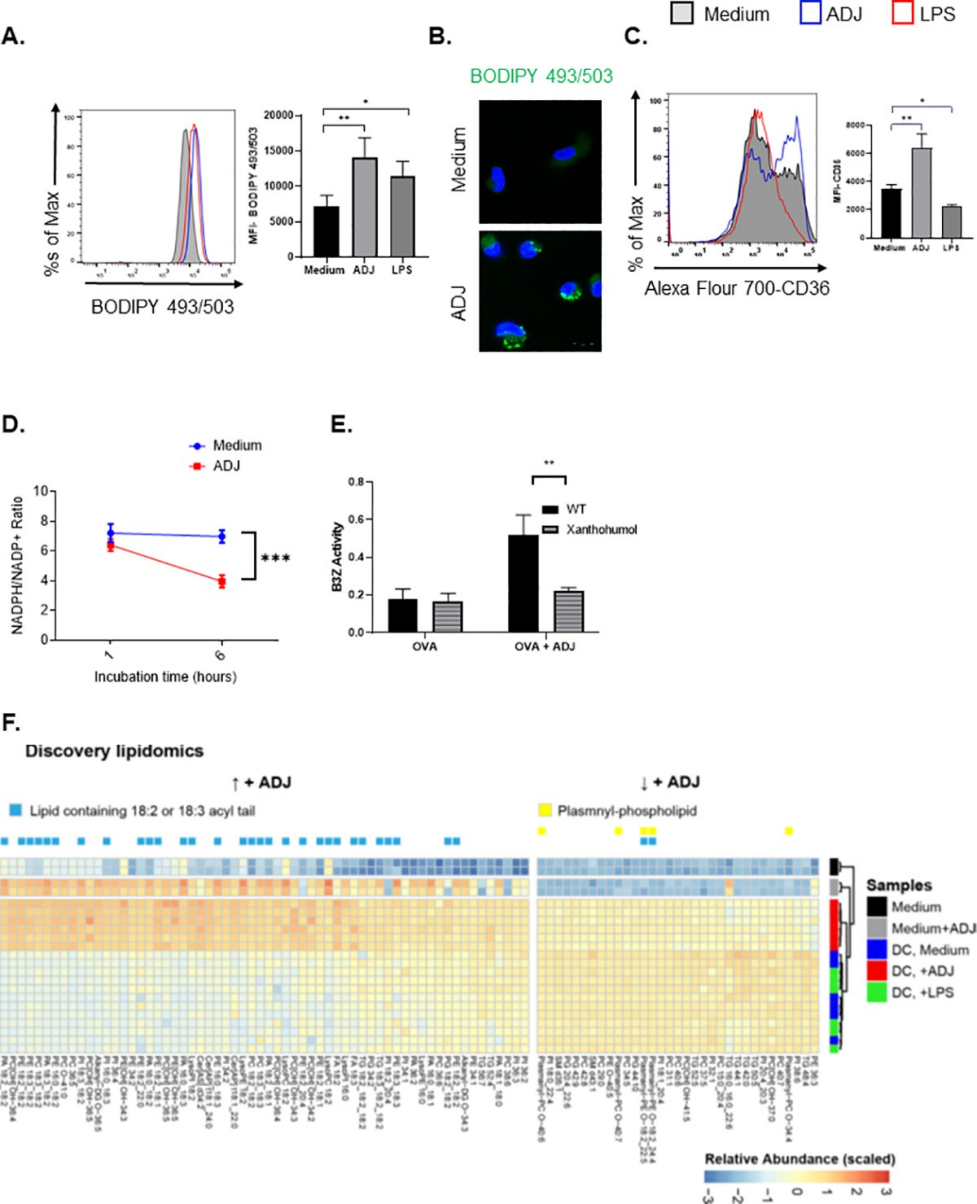
Carbomer-based adjuvant induces intracellular lipid body formation and alters intracellular lipidomes in DCs. (A) Quantification of neutral lipid droplets by flow cytometry using BODIPY 493/503 dye. BMDCs treated with ADJ (1%) for 24 h were stained with BODIPY 493/503 dye and analyzed by flow cytometry (B) Confocal microscopy of DCs were cultured with ADJ (1%) for 24 h, stained with BODIPY 493/503, and counter-stained with DAPI. Scale bars, 10 μm. (C) FACS analysis of CD36 expression in unstimulated, and ADJ- or LPS-stimulated DCs after 24 h. (D) NADPH/NADH+ ratio in resting, ADJ- or LPS-stimulated DCs. (E) β-galactosidase production by B3Z cells after co-culture with BMDCs pre-treated with OVA ± ADJ in the presence/absence of xanthohumol for 5 h. (F) Heat-map of lipids from media or DCs stimulated with ADJ ± LPS; *P* < 0.05 (ANOVA for +ADJ treatment, Tukey post-hoc corrected p-values) and abundance was scaled across samples. Lipids that increased with ADJ treatment were enriched for 18:2 or 18:3 acyl-chains (blue bar). Lipids that were decreased with ADJ treatment contained more plasmanyl-phospholipids (yellow bar). Abbreviations: TG, triglyceride; DG, diglyceride; FA, fatty acid; PA, phosphatidic acid; PC, phosphatidylcholine; PE, phosphatidylethanolamine; PG, phosphatidylglycerol; PI, phosphatidylinositol; SM, sphingomyelin; Cer [AP], alpha-OH fatty acids with pytosphingosine; Cer [AS], alpha-OH fatty acids with sphingosine. Data are representative of 2 independent experiments. Error bars show SEM; *P<0.01; **P<0.001; ***P<0.0001 (One-way ANOVA- A, C; Student’s t-test- D-E).

Next, we explored how ADJ might enhance cross-presentation independent of aerobic glycolysis in DCs. The formation of intracellular lipid bodies (LB) was shown to be critical for efficient DC cross-presentation [60,61]. Therefore, we determined whether LBs are also essential for ADJ-mediated cross-presentation. To assess whether ADJ induced intracellular formation of LBs in DCs, unstimulated, LPS-, or ADJ-treated DCs were stained with BODIPY 493/503 which stains neutral lipids. Neutral lipids were barely detected in resting DCs, but ADJ or LPS-stimulated DCs contained abundant levels of neutral lipids, as indicated by an increase in MFI of BODIPY 493/503 in a flow cytometer or as visualized by confocal microscopy **(Fig 6A and 6B)**. To elucidate mechanisms underlying the increased intracellular content of neutral lipids in DCs, we examined whether ADJ altered FA uptake by promoting expression of the scavenger receptor CD36. Intriguingly, ADJ increased the expression of CD36 in ADJ-stimulated DCs, but LPS downregulated CD36 expression in DCs **(Fig 6C).**

It has been reported that the glucose-derived pentose phosphate pathway (PPP), an offshoot of the glycolytic pathway, is critical for generation of LBs upon TLR-stimulation in DCs [49]. We did not observe increased glycolytic flux in ADJ-stimulated cells to fuel PPP **(Fig 5I)**, but we hypothesized that the NADPH/NADP+ ratio will be altered in ADJ-treated cells because imported FAs need to be activated before incorporation into triglycerides by esterification with coenzyme A, through a reaction catalyzed via fatty acyl-CoA synthetase. As an indirect measure of NADPH/NADP+ ratio, we quantified optical redox ratio (NAD(P)H/NAD(P)H + FAD ratio) in ADJ-stimulated cells using optical multiphoton microscopy [62]. We discovered a significant drop in the redox ratio in ADJ-stimulated cells compared to unstimulated cells **(S6 Fig)**. To further distinguish NADPH/NAD+ from NADH/NAD+ ratio, we used a bioluminescence-based assay and confirmed that ADJ-stimulated cells displayed lower NADPH/NADP+ ratio **(Fig 6D)**. Together, these data suggested that ADJ stimulation likely induced LB formation by utilizing intracellular NADPH and imported FAs, independent of glucose-derived *de novo* FAs.

Lastly, to examine whether LB formation is required for ADJ-driven cross-presentation, we treated DCs with xanthohumol, a DGAT1/2 inhibitor. Notably, treatment of ADJ-treated DCs with xanthohumol significantly inhibited cross-presentation and activation of B3Z cells by ADJ-treated DCs **(Fig 6E)**. Together, this suggested that LBs might be crucial for ADJ-induced DC cross-presentation.

### ADJ alters intracellular lipidome in DCs and increases accumulation of 18:2 and 18:3-containing lipids

The saturation and oxidation status of lipids might modulate cross-presentation by DCs [63–66]. However, how lipid composition governs DC cross-presentation remains unknown. In order to map changes in lipid species in ADJ-treated cells, we employed a discovery lipidomics mass spectrometry approach. With this approach, we identified 446 unique lipid species in ADJ-treated DCs as compared to resting or LPS-treated DCs, and ADJ-treated DCs displayed significant increases in lipids containing acyl-chains with linoleic (18:2) or alpha-linoleic (18:3) acid (**Fig 6F**), which appear to be constituents of the adjuvant ADJ (**Fig 6F,** gray bars). Note that media with ADJ also contained an increased abundance of 18:2 and 18:3-containing lipids, as compared to media alone. In addition, ADJ treatment also led to increases in ceramides containing alpha-hydroxy fatty acids (Cer[AP] and Cer[AS]) and decreased abundance of plasmanyl-phospholipids. Hence, these lipidomic profiles further highlight the global lipid changes induced by ADJ treatment, characterized by increased abundance of linoleic and alpha-linoleic acyl-chains within phospholipids and triglycerides, likely resulting in changes in membrane fluidity. Together, our data suggested that changes in lipid composition could be critical for ADJ-mediated cross-presentation in DCs.

## Discussion

In this manuscript, we report that a carbomer-based nano-emulsion adjuvant, ADJ, promoted memory T cell-dependent protective immunity against intracellular pathogens, *Listeria monocytogenes* and vaccinia virus. Consistent with strong induction of CD8 T-cell responses to subunit antigens *in vivo*, ADJ promoted DC cross-presentation *in vitro*. Mechanistic investigations of antigen processing and the metabolic basis for adjuvant action demonstrate that ADJ promoted multiple aspects of antigen cross-presentation in DCs, in the apparent absence of switch from catabolic to anabolic metabolism. This constitutes a novel mechanism because the current axiom suggests that engagement of active aerobic glycolysis in DCs is necessary for their activation and ability to stimulate CD8 T cells [18].

How did ADJ enhance DC cross-presentation and potentiate CD8 T cell immunity *in vivo*? Effective cross-presentation required antigen targeting to less acidic intracellular compartments, slow degradation of antigens by proteases, and translocation of endosomal antigens into cytosol. Intriguingly, we discovered that ADJ-treated DCs contained greater amounts of degraded antigens, compared to untreated DCs, but this was not linked to elevated antigen uptake. Increased antigen cross presentation mediated by ADJ appears to be the result of greater induction of ROS by ADJ that presumably leads to alkalization, attenuated antigen degradation, and accumulation of partially degraded antigens in alkaline endosomes. Thus, one mechanism by which ADJ might enhance cross-presentation is by delaying antigen degradation and promoting partially degraded antigen accumulation in DCs, which in turn sustains antigen presentation by DCs.

Our studies also demonstrated that ADJ-treated DCs utilized an endosomal-to-cytosol pathway of cross-presentation, as indicated by augmented endosomal protein leakage into cytosol and abolishment of ADJ-driven cross-presentation by pharmacological inhibition of cytosolic proteasomes. The molecular mechanisms underlying the translocation of endosomal antigens into cytosol during cross-presentation remains controversial. One possible mechanism is explained by the transporter hypothesis, in which exogenous antigens are unfolded by gamma-interferon-inducible lysosomal thiol reductase (GILT) in the phagosome and transported into cytoplasm by ER-associated degradation (ERAD) machinery or chaperone-mediated transport by Hsp90 [67–69]. Another potential mechanism is endosomal membrane disruption, induced by NOX2-dependent ROS production that serves as precursors for lipid peroxides in the endosomes. In our studies, we found that: (1) ADJ strongly induced ROS production; (2) NOX2 deficiency and pharmacological inhibition of NOX2 assembly abolished cross-presentation; (3) antioxidant tocopherol diminished ADJ-driven cross-presentation. Therefore, our data favor the endosomal disruption model, where ADJ-induced ROS might have a dual function in cross-presentation: increase endosomal pH to delay antigen degradation and promote antigen translocation into cytosol by lipid peroxidation.

Only recently, it was discovered that TLR-driven metabolic shift to anabolic metabolism is an integral component of the activation program that is required to activate naïve T cells [18]. While the metabolic basis of how TLR agonists support DC effector functions is well characterized, the exact metabolic roles of many immune adjuvants in dictating DC activation and antigen presentation remain poorly understood. Our data suggest that the two important energy yielding metabolic pathways, glycolysis and OXPHOS, were minimally engaged or inactive in ADJ-treated DCs, which is indicative of a unique cellular state of metabolic quiescence during cross-presentation. How ADJ-treated DCs effectively stimulate T cells *in vivo* without the need to switch to glycolysis or to trigger enhanced mitochondrial metabolism remains unclear. Recently, it was reported that DCs contain intrinsic glycogen, which can be readily catabolized into glucose upon LPS stimulation to fuel intracellular glucose [70]. Unlike FAO, which requires a substantial number of functional mitochondria, glycogenolysis occurs in the cytoplasm. Because ADJ treatment results in a loss of mitochondrial functions and minimal engagement of glycolysis in DCs, it is plausible that ADJ-treated DCs can catabolize intrinsic glycogen into glucose to sustain their survival in the absence of functional mitochondria. Follow-up studies should evaluate whether ADJ-treated DCs utilize intracellular glycogen reserves during metabolic quiescence and/or whether glycogenolysis is required for ADJ-mediated DC cross-presentation.

Despite a ‘hypometabolic’ state, ADJ-treated DCs displayed effective antigen cross-presentation and stimulated CD8 T cell responses *in vivo*. However, this leads to the question of why ADJ-stimulated DCs adapt to this particular type of metabolism that is metabolically inefficient, at least in terms of ATP production. A recent study suggests that high levels of glucose represses DC-induced T cell responses by engaging the mTORC1-HIF1α/iNOS pathway in DCs [71]. In their studies, limiting glucose availability to DCs enhanced T cell responses by diminishing competition for glucose from DCs in the immune microenvironment. It is plausible that ADJ drives DCs to a metabolic state that is less dependent upon glucose-driven catabolic pathways, which enables DC outputs tailored for effective cross-presentation to T cells. For instance, conventional processing of exogenous antigens involves acidification of the lysosomal compartment, which is required for efficient generation of peptides for loading into MHC II and that is dependent on activity of ATP-driven proton pumps [72]. During this process, lysosomal V-ATPase complex is assembled by intracellular H^+^ ions generated by aerobic glycolysis (resulting from oxidation of NADH into NAD^+^ and H^+^ ions), leading to an increase in lysosomal acidity. Notably, ADJ did not trigger redox imbalance of NADH/NAD+ ratio in DCs, but only reduced intracellular ATP production. Thus, it is plausible that maintenance of a low metabolic state by reducing a key carbon source for generating ATP and keeping balanced redox ratio might be essential for reducing intracellular acidity by inhibiting lysosomal V-ATPase assembly in DCs.

The divergent functions of inflammasome activation in shaping adaptive immunity have been demonstrated for other vaccine adjuvants, such as Alum and ISCOM [54]. Production of IL-1β and IL-18 suggest that ADJ might activate inflammasomes in DCs. This finding was unexpected, especially because LPS-induced succinate stabilized HIF-1α in macrophages, which is known to be required for inflammasome activation under inflammatory conditions [73]. However, ADJ did not promote stabilized-HIF1α accumulation under normoxia in DCs. It has been also documented that phago-lysosomal destabilization after adjuvant phagocytosis, such as Alum and Carbopol, is an important step in inflammasome activation [74,75]. While we have not directly characterized the intracellular location of ADJ in DCs, ADJ increased lysosomal pH, which in turn may result in lysosomal stabilization. Inflammasome activation has been closely associated with enhanced cross-presentation in multiple studies [34,35], but our studies showed that loss of NLRP3, ASC or caspase-1 did not affect the ability of ADJ to enhance DC cross-presentation *in vitro*. It is possible that ADJ might stimulate IL-1β by inflammasome-independent mechanisms [76–78] and/or that ADJ modulates DC cross-presentation, by inflammasome-independent mechanisms, at least *in vitro*. Caspase 1 deficiency did not impair ADJ’s ability to induce DC cross-presentation *in vitro* and *in vivo*, indicating that ADJ likely engages other mechanism(s) needed for DC cross-presentation, without caspase-1 activity. This finding is consistent with a previous report, in which induction of OVA-specific antibodies by Carbopol, a polyanionic carbomer, was not significantly affected in caspase 1-deficient mice [74]. Future studies are warranted to determine how and whether ADJ triggers inflammasome activation in DCs and the role of inflammasome activation in engendering protective cell-mediated immunity *in vivo*.

The causative link between the formation of intracellular LBs and cross-presentation efficiency by IFN-γ and ISCOMs has been recently established [60,61]. Consistent with previous findings, our studies also suggest that ADJ-induced cross-presentation requires intracellular LB formation in DCs. However, how LBs exert their effects on DC cross-presentation remains undetermined. While we have not yet examined whether ADJ-aided LBs directly affect antigen processing, our lipidomic studies highlight the unique composition of lipids within ADJ-treated DCs, which shows accumulations of 18:2 and 18:3 acyl tails and ceramides, presumably due to uptake of ADJ itself. Because ADJ upregulates CD36 expression in DCs, it is conceivable that ADJ enhances the uptake of external lipid without *de novo* fatty acid synthesis derived from glucose [79]. In line with low metabolic profiles in ADJ-stimulated DCs, the formation of intracellular LBs using external fatty acids could be a more efficient pathway to store intracellular lipids since it requires minimal energy and overall metabolic activities. The global intracellular lipidome modified by ADJ suggests a possible explanation for ADJ-induced cross-presentation, presumably by regulating antigen export to cytosol during cross-presentation. For example, an increase in double-bonds within phospholipids could directly increase membrane fluidity, leading to an increase in endosomal antigen leakage [80,81]. An enrichment in certain ceramides could also contribute to the formation of lipid rafts, which are known to be critical for regulation of endosomal NOX2 assembly [82]. Thus, enrichment of certain lipid classes in the endosomes mediated by ADJ, such as unsaturated phospholipids and ceramides, could be another important rate-liming step for antigen export to the cytosol, in addition to ROS-driven endosomal disruption.

In summary, we have identified a carbomer-based adjuvant (ADJ) that elicits protective CD8 T cell responses to soluble subunit antigens, protects against viruses and an intracellular bacterial pathogen, and enhances cross-presentation by the cytosolic pathway. The most striking finding is that ADJ-stimulated DCs, which are highly efficient in cross-presenting antigens, exhibited a distinct metabolic state that is characterized by minimum glycolytic activity, low mitochondrial respiration, and intracellular LB formation (**S7 Fig**). Thus, our model challenges the prevailing metabolic paradigm by suggesting that retaining DCs in a quiescent state is a unique mechanism to regulate efficient DC cross-presentation. Our findings have significant implications in understanding the mechanism of action of adjuvants and development of safe and effective vaccines that elicit potent T cell-based immunity against infectious diseases, such as AIDS, TB and malaria.

## Methods

### Ethics statement

All animal experiments were performed in accordance with the protocol (Protocol number V5308 and V5564) approved by the University of Wisconsin School of Veterinary Medicine Institutional Animal Care and Use Committee (IACUC). The animal committee mandates that institutions and individuals using animals for research, teaching, and/or testing much acknowledge and accept both legal and ethical responsibility for the animals under their care, as specified in the Animal Welfare Act (AWA) and associated Animal Welfare Regulations (AWRs) and Public Health Service (PHS) Policy.

### Experimental animals

7-12-week-old C57BL/6J (B6), Gp91 (NOX2) -/- (Stock number, 002365), and TAP1 -/- (Stock number: 002944) were purchased from Jackson Laboratory or obtained from restricted-access SPF mouse breeding colonies at the University of Wisconsin-Madison Breeding Core Facility. Caspase 1-deficient, ODD-LUC mice backcrossed to Albino C57BL/6 background, OT-I mice were provided by Drs. J. D. Sauer, Richard Eisenstein, and Jing Zhang (University of Wisconsin-Madison), respectively. KLF2-GFP reporter mice were provided by Dr. Stephen Jameson (University of Minnesota). NLRP3-KO and ASC-KO mice were kindly provided by Drs. Vishva Dixit and Kim Newton (Genentech-Roche, CA); NLRP3/AIM2-dKO mice were kindly provided by Dr. Thirumala-Devi Kanneganti (St. Jude Children’s Hospital, TN).

### Chemicals and reagents

Adjuplex (endotoxin-free) was purchased from Empirion LLC. Hen egg white ovalbumin grade V (OVA) from chicken egg white (A5503), LPS purified from Escherichia coli O111:B4 (L2630), Leupeptin (L2884), α-Tocopherol (T3251), gelatin from cold water fish skin (G7041), Collagenase B (11088831001), Collagenase D (11088882001), and Cytochrome c from equine heart (C2506) were purchased from Millipore Sigma. Ovalbumin, Alexa Fluor 647 Conjugate (O34784), DQ Ovalbumin (D12053), CellROX Deep Red reagent (C10422), Lysotracker Deep Red reagent (L12492), LiveDead eFlour 780 (A10628), BODIPY 493/503 (D3922), MitoTracker Deep Red FM (M22426), DiOC_6_ (D273), MitoSOX Red Mitochondrial Superoxide Indicator (M36008), Protease Inhibitor Tablets (PI88666), Pierce Phosphatase Inhibitor Mini Tablets (88667), Thermo Scientific Halt Protease Inhibitor Cocktails (78430), Prolong Gold Antifade (P36934), and DAPI (D1306) were purchased from Thermo Fisher Scientific. pHAb Amine Reactive Dye (G9841) was purchased from Promega. Epoxomycin (10007806) and xanthohumol (15399) were purchased from Cayman Chemicals. Paraformaldehyde was purchased from Electron Microscopy Sciences (15710-S). Chlorophenol red-β-D-galactopyranoside (CPRG, sc-257242) and apocynin (sc-203321) were purchased from Santa Cruz Biotechnology. NP-40 (M158-50ML), Tris (97061–794), Tween-20 (97062–332), Triton X-100 (97063–864), Glycine (0167-1kg), and brain heart infusion (90003–040) were purchased from VWR (Amresco). Glutaraldehyde (O2957-1) and Agar (BP1423-500) were purchased from Fisher Scientific.

### Antibodies

Hamster anti-CD11c-BV421-conjugated (N418, 565452), Hamster anti-CD11c/PE-Cy7-conjugated (N418, 558079), Rat anti-CD40-BV421-conjugated (3/23, 562846), Hamster anti-CD80-APC-conjugated (16-10A1, 560016), Mouse anti-H-2K^b^-FITC-conjugated (AF6-88.5, 562002), Mouse anti-I-A^[b]^-PE-CF594-conjugated (AF6-120.1, 1:400, 562824), Annexin V-PE-conjugated (556421), Rat Anti-CD4-BUV496-conjugated (GK1.5, 564667) Rat Anti-CD8-BUV395-conjugated (53–6.7, 563786), Rat Anti-CD44-BV510-conjugated (IM7, 563114), and BV421 Streptavidin (563259) were purchased from BD Biosciences. Rat anti-CD86-PE/Cy7-conjugated (GL-1, 105014) was purchased from Biolegend. Rat anti-CCR7-PE-conjugated (4B12, 12-1971-82), Hamster anti-CD36-Alexa Fluor 700-conjugated (HM36, 56-0362-80), and Biotin Mouse OVA257-264 (SIINFEKL) peptide bound to H-2K^b^ Monoclonal Antibody (1:50, eBio25-D1.16 (25-D1.16), 13-5743-81) were purchased from eBioscience. APC-conjugated H2-K^b^ tetramers bearing the ovalbumin peptide SIINFEKL was obtained from NIH Tetramer Core Facility at Emory University. Mouse anti-NOS2 Antibody PE-conjugated (C-11, sc-7271) and Rat anti-mouse LAMP1 antibody (1D4B, sc-19992) were purchased from Santa Cruz Biotechnology. Purified Anti-Mouse CD16 / CD32 (Fc Shield, 70-0161-U500, 2.4G2, 1:100) was purchased from Tonbo Biosciences. Alexa Fluor 488 goat anti-rabbit IgG (H+L) secondary antibodies (A11008), Alexa Fluor 568 goat anti-rat IgG (H+L) secondary antibodies (A11077), and goat anti-rabbit IgG (H+L) Cross-Adsorbed Secondary Antibody, HRP (G-21234) were purchased from Thermo Fisher Scientific. Rabbit anti-EEA1 antibody (C45B10, 3288), Rabbit anti-Phospho-Akt (Ser473, Clone: D9E) antibody, Rabbit anti-Akt polyclonal antibody (9272S), Rabbit anti-phosphor-p70 S6 kinase (Thr389, Clone: 108D2), and Rabbit polyclonal anti-p70 S6 kinase antibodies (9202S) were purchased from Cell Signaling Technology

### Tissue processing and flow cytometry

Primary monoclonal antibodies for detecting surface markers/tetramers were used for flow cytometry at 1:200 dilution (provided in the resource table) unless stated otherwise. Spleens and lymph nodes were processed into single-cell suspensions by standard procedures. In some experiments, lymph nodes and lungs were digested in 2mg/mL of Collagenase (Collagenase B for lungs; Collagenase D for draining lymph nodes) for 15 minutes at 37C. Single-cell suspensions were first stained for viability with LiveDead eFlour 780 stain (eBioscience), blocked with FACS buffer (1% BSA in PBS) containing FC block (1:100), and stained with antibodies diluted in Brilliant Stain Buffer (BD Biosciences) or FACS buffer for 30–60 minutes. To detect K^b^/SIINFEKL expression, DCs were first stained with biotin-labeled anti- SIINFEKL/H-2K^b^ monoclonal antibody on ice for 40 minutes. Next, samples were stained with BV421-streptavidin (1:500) on ice for 30 minutes. Intracellular staining of iNOS was performed using Cytofix/Cytoperm fixation/permeabilization kit (BD Biosciences; 555028) as previously described [51]. Samples were acquired with a BD LSR Fortessa (BD Biosciences) and data were analyzed with FlowJo software (TreeStar, Ashland, OR).

### BMDC generation and cell culture

Primary cultures of bone marrow-derived DCs were generated as previously described [83,84]. Briefly, femur, tibia, and humerus were flushed using RPMI-1640 medium supplemented with 1% FBS. Bone marrow cells were cultured in RPMI-1640 medium supplemented with 10% fetal bovine serum, 100 U/ml penicillin G, 100 ug/ml streptomycin sulfate, and 10ng/ml GM-CSF (Peprotech) in 150mm petri dishes. Equal parts of additional media with 10ng/ml of GM-CSF were added on day 3. Loosely attached immature DCs were collected at day 6–7 and subsequently used for the experiment. The B3Z hybridoma was a generous gift from Dr. Bruce Klein (University of Wisconsin-Madison). B3Z cells were maintained in Iscove’s Modified Dulbecco’s media supplemented with 10% FBS, 100 U/ml penicillin G and 100 g/ml streptomycin sulfate. Vero cells were maintained in low-glucose DMEM media, supplemented with 10% FBS, 100 U/ml penicillin G and 100 g/ml streptomycin sulfate

### Vaccination, viral titers, and enumeration of Listeria challenge

C57BL/6J mice were vaccinated by the subcutaneous route at the tail base with 50ul of the vaccine (containing 5% ADJ and 10ug chicken OVA). Mice were boosted after 21 days of the initial vaccination. At > 40 days after vaccination, mice were challenged intranasally with 2 X 10^6^ recombinant vaccinia virus-expressing OVA as previously described [29]. Spleens, lungs, and ovaries were used for viral titers. Tissue samples for viral titers were homogenized in 400ul 1mM pH 8.0 Tris using bead homogenizer. Clarified supernatant was trypsinized in Trypsin-EDTA (0.05%) and titrated in complete RPMI on Vero cells (American Type Culture Collection: ATCC CCL-81). *Listeria monocytogenes* expressing chicken ovalbumin (LM-OVA) was provided by Dr. Hao Shen (University of Pennsylvania School of Medicine) as previously described [30]. Mice were infected intravenously with 1.7 x 10^5^ CFUs of LM-OVA. To quantify Listeria burdens, tissues were homogenized in GentleMACS C-Tubes via GentleMACS dissociator. Organs were processed in sterile 0.1% Nonidet-P40 + PBS in GentleMACS C Tubes. Serial dilutions of tissue samples were plated on brain heart infusion (BHI) agar plates for 24 hours at 37 C’. Vaccinia viral titer and Listeria burden in tissues were normalized by the weight of the tissues.

### *In vivo* KLF2-GFP detection and DC-T cell priming

To assess *in vivo* KLF2-GFP expression, cDCs were analyzed in draining lymph nodes 24 hours after footpad injection with 25ul of the vaccine (containing 10ug chicken OVA with or without 10% ADJ or 10ug LPS). For *in vivo* DC-T cell priming, BMDCs were loaded with 1mg of OVA with or without 1% ADJ for 6 hours, washed twice with PBS, and injected intravenously into mice. Spleens were collected on day 8 and the percentages of CD44^HI^ H-2K^b^/SIINFEKL tetramer-binding CD8 T cells were quantified by flow cytometry.

### OT-I CD8 T cell *in vitro* proliferation

OT-I CD8 T cells from the spleen were purified using mouse Pan T cell isolation (Miltenyi biotec) and fluorescently labeled with CellTrace CFSE Cell Proliferation Kit (Thermo Fisher Scientific) according to manufacturer’s instructions. BMDCs (1x10^5^ cells/well) were cultured with OVA or ADJ+OVA on 96 well flat bottom plate (Corning), extensively washed with sterile PBS, and co-cultured with purified CFSE-labeled OT-I CD8 T cells (5x10^5^ cells/well) for 72 hours. After 72 hours, OT-I CD8+ T cell division was measured by CFSE dilution using flow cytometry.

### B3Z activation assay for *in vitro* cross presentation

The cross-presentation capacity of murine BMDCs was measured using B3Z hybridoma cells, as previously described [33,85]. Briefly, DCs were plated at 1 x 10^5^ cells/well in 96-well round-bottom culture-treated plates (Corning). In some experiments, BMDCs were pre-treated for 1 hour with aponocyin (300μM) leupeptin (50μM), α-tocopherol (100μM), epoxomicin (10μM) or xanthohumol (30μM). Subsequently, BMDCs were cultured with OVA (1mg/ml) with or without ADJ and appropriate chemicals for 5 hours. Next, BMDCs were fixed with 0.025% glutaraldehyde for 2 minutes at room temperature. washed with PBS and cultured with B3Z cells (1 x 10^5^ cells/well) for 18 hours. After 18 hours, B3Z cells were washed and incubated with CPRG substrate (0.15mM) in 200ul of lysis buffer (0.1% NP 40+ PBS) for 18 hours at room temperature. The absorbance at 590nm was measured using a plate reader. Wells containing B3Z cells + BMDCs without OVA served as background control.

### Cytokine detection from cell culture supernatants

Supernatants from cultures of BMDCs cultured with or without ADJ (1%) were collected at 24 hours. Cytokines in cell culture supernatants were quantified using Bio-Plex Pro Mouse Cytokine 23-plex Assay (Bio-Rad, M60009RDPD), IFN-β ELISA kit (R&D Systems, DY8234-05), and IL-18 ELISA (Thermo Fisher Scientific, BMS618-3) according to manufacturer’s protocol.

### Antigen capture and processing assay

BMDCs were seeded at 4–5 x 10^5^ cells per well in 96-well culture flat-bottom plate (Corning) for the following assays. For antigen uptake assay, 20ug Alexa Fluor 647-conjugated OVA was mixed with or without ADJ (1%) in 200ul of complete RPMI media. BMDCs were incubated with pre-warmed Alexa Fluor-647 OVA with or without 1% ADJ for 10 or 30 minutes. The pH of OVA-containing endosomal compartment was determined using ovalbumin-conjugated with pHAB Amine Reactive dye, as previously described with some modifications [86,87]. Conjugation of ovalbumin with pHAB-amine reactive dye was performed according to manufacturer’s instructions. Briefly, OVA was labeled with amine-reactive dye at 1:10 excess molar ratio (ovalbumin:pHAB amine reactive dye). Free-unlabeled dye was removed by PD-10 columns (GE healthcare) and concentrated using Amicon Ultra Centrifugal Filters (Millipore). BMDCs were incubated with pHAB-conjugated OVA with or without 1% ADJ for 30 minutes, and chased for 30 minutes. Antigen degradation studies were performed using DQ-OVA; DCs were incubated with DQ-OVA (20ug) with or without 1% ADJ for 30 minutes, and chased for 6 hours.

### Detection of intracellular ROS, H_2_O_2,_ lipid peroxidation, and intracellular acidity

ROS and H_2_O_2_ productions were measured using CellROX Deep Red reagent and ROS-Glo H_2_O_2_ assay, respectively. For ROS production, samples were washed with PBS and incubated with 50nM CellROX Deep Red in PBS for 15 minutes at 37C. For intracellular H_2_O_2_ detection, ROS-Glo H_2_O_2_ assay (Promega; G8820) was performed according to manufacturer’s instructions; luciferin activity from added H_2_O_2_ substrate was measured by SpectraMax i3x (Molecular Probes). Lipid peroxidation was measured using Image-iT lipid peroxidation kit (Thermo Fisher Scientific: C10445) according to manufacturer’s instructions. For tracking acidic organelles, samples were washed and incubated with 50nM Lysotracker Deep Red in PBS for 15 minutes at 37C.

### Endosomal antigen leakage assay

Cytochrome C release assay was performed as previously described, with minor modifications [43]. Briefly, BMDCs were cultured with 5mg/ml of equine cytochrome C (Sigma Aldrich) with or without 1% ADJ. After 24 hours, cells were stained with Annexin-V and analyzed by flow cytometry.

### 20S proteasome activity assays

In BMDCs, the activities of proteasomes containing luminogenic substrates (the Suc-LLVY, Z-LRR and Z-nLPnLD sequence recognized by the 20S proteasome) were measured by Proteasome-Glo Chymotrypsin-Like, Trypsin-Like and Caspase-Like Cell-Based Assays (Promega, G1180), according to manufacturer’s instructions [88].

### Immunofluorescence, confocal laser scanning microscopy for co-localization

For co-localization of antigen-containing compartment, DCs were seeded in 24-well plates at a density of 1x 10^6^ cells/well on 1.5mm coverslips (Warner Instrument) and cultured in complete phenol-red free RPMI 1640 media (Lonza) for 30 minutes at room temperature. DCs were pulsed with Alexa Fluor 647-OVA (40 ug/ml) +/- ADJ (1%) for 15 minutes at 37 C, and chased in complete phenol-red free RPMI media for 20 or 60 minutes at 37 C. Cells were washed with PBS, fixed with 4% paraformaldehyde (Electron Microscopy Services) at room temperature for 20 minutes, permeabilized using 0.2% Triton X-100 and blocked in blocking buffer (10% goat serum, 0.1% cold-fish gelatin, 0.1% tween-20) for 1 hour. Antibodies (rabbit anti-EEA1 (Clone: C45B10, 3288S, 1:50, Cell Signaling technology), rat anti-LAMP1 (Clone: 1D4B, sc-19992, 1:50, Santa Cruz Biotechnology) were diluted in blocking buffer and used to detect early endosomes or lysosomes at 4C’ overnight:, Coverslips were extensively washed with PBS + 0.1% Tween-20 and incubated with Alexa 564-conjugated rat IgG antibody or Alexa 488-conjugated rabbit IgG antibody (Invitrogen) in blocking buffer at room temperature for 1 hour. Samples were counter-stained with DAPI (Invitrogen), washed, mounted with Prolong Gold Anti-fade mountant (Invitrogen) and imaged on a Leica SP8 confocal laser-scanning microscope at 63X objective lens. The degree of co-localization was calculated using LAX-S Software (Leica).

### Metabolism assays

For real-time analysis of ECAR and OCR, Glycolysis Stress and Mito Stress tests (Agilent: 103020–100 and 103708–100) were performed according to manufacturer’s instructions [89]. BMDCs were analyzed with an XF-96 Extracellular Flux Analyzer (Seahorse Bioscience). 1–2 x 10^5^ cells/well were seeded in 96-well tissue-culture treated flat-bottom plates for the metabolic measurements; ATP concentrations, glucose, lactate, NAD+/NADH, and NADP+/NADPH were quantified using CellTiter-Glo, Glucose-Glo, Lactate-Glo, NAD+/NADH-Glo, and NADP+/NADPH-Glo kits (Promega: G9241, J6021, J5021, G9071, and G9081), respectively, according to manufacturer’s instructions. For accurate quantification of metabolites from the cell culture supernatant, dialyzed FBS (Gibco; A3382001) was used in red phenol-free complete RPMI media. Nitrite levels were quantified using Griess’ reagent (Sigma Aldrich; 03553-100ML). 3–4 x 10^5^ cells/well were used in 96-well non-treated flat-bottom plates for the following metabolism assays and transferred to 96-well round-bottom plates for staining; mitochondrial contents, membrane potentials, and mitochondrial ROS were measured by treating cells with Mitotracker Deep Red, DiOC_6_, and MitoSOX, respectively in PBS for 30 minutes according to manufacturer’s protocol. To visualize neutral lipids, cells were stained with 500 ng/ml BODIPY 493/503 in PBS for 15 minutes; stained unfixed cells were acquired immediately on a flow cytometer.

### KLF2-GFP and ODD-luc reporter assay

BMDCs derived from ODD-Luc or KLF2-GFP mice were cultured with ADJ (1%) or LPS (100ng/ml) For KLF2-GFP reporter experiments, samples were washed and immediately acquired on flow cytometer. For ODD-luc DCs, cells were lysed using passive lysis buffer and luciferase activities were measured using Luciferase reporter assay system (Promega; E4030) according to manufacturer’s instructions.

### Quantification of the optical redox ratio by live cell microscopy

Optical imaging of BMDCs was performed as described previously [62]. BMDCs were seeded at a density of 1 x 10^6^/ ml in glass bottom dishes 2 hours before imaging. For the time course experiments, 1% ADJ was added immediately before imaging. Cells were imaged in a stage-top incubator maintained at 37°C with CO_2_ supplementation. Four locations within each dish were imaged for control and ADJ-treated groups. The optical redox ratio was calculated from intensity images, which were acquired for 60 seconds as previously described [62]. The total number of NADH photons was divided by the sum of the total number of NADH photons and the total number of FAD photons on a single pixel basis. A custom Cell Profiler pipeline was used to threshold out nuclear signal and background. The average redox ratio value per cytoplasm was then computed.

### Immunoblot

BMDCs that were cultured with or without ADJ (1%) or LPS (100ng/ml) were washed with cold PBS and lysed in RIPA buffer (CST) containing protease and phosphatase inhibitor cocktail. Total protein levels in each lysate were estimated using Pierce BCA protein assay kit (Thermo Scientific; 23227). Samples containing 30-40ug protein were loaded and resolved on a 12% SDS-PAGE gel (Genscript), transferred to PVDF membrane using iBlot 2 Gel Transfer (Thermo Fisher Scientific; IB24001), blocked with 5% BSA in TBST for phospho-proteins and 5% milk for total proteins in TBST for 1 hour and probed with primary antibodies (at 4C overnight). Primary rabbit antibodies were used as follows at 1:1000 dilution in 5% BSA in TBST: rabbit monoclonal anti-phospho-Akt (Ser473; Clone: D9E), rabbit polyclonal anti-total Akt, rabbit monoclonal anti-phosphor-p70 S6 kinase (Thr389, Clone: 108D2), and rabbit polyclonal anti-p70 S6 kinase antibodies. Blots were extensively washed with TBST and incubated with goat anti-rabbit IgG (H+L)-HRP antibodies (Thermofisher) diluted in 5% non-fat milk in TBST for 1 hour at room temperature. Protein bands were visualized by ECL prime western blotting detection reagent (GE Healthcare.) Membranes were stripped with mild stripping buffer (0.1% SDS, 0.1% Tween 20, 1.5% Glycine), when necessary.

### Discovery lipidomics by LC-MS

Samples were spun down and snap frozen in liquid N_2_ and stored at -80C until extraction. Media samples were thawed on ice, and 50ul of media was transferred to microcentrifuge tube; cell pellets were directly extracted in tubes. To perform extraction, 187.5μL of chilled methanol and 750μL of chilled methyl tert-butyl ether (MTBE) was added to each tube, followed by the addition of 5mm stainless steel beads. All samples were then bead-beaten in a cold room on a Retsch MM400 mixer mill at a frequency of 25 cycles/sec for 5 minutes to complete cell lysis. This mixture was then vortexed for 1–2 s and quickly centrifuged to remove any stray droplets from the tube openings. Then 187.5μL of chilled water was added to each tube to separate the hydrophobic and hydrophilic compounds into separate phases. All samples were then vortexed for 30 seconds and then centrifuged at 4C for 2 minutes at a speed of 14000 x g to re-pellet any cell debris. A total of 300μL of the top-layer of the biphasic extraction was removed from the tube and collected into a low volume borosilicate glass autosampler vial with tapered insert and dried by vacuum concentrator. All samples were reconstituted with 50μL of a 9:1 MeOH:Toluene solution for injection.

LC-MS analysis was performed on an Acquity CSH C18 column held at 50°C (100 mm x 2.1 mm x 1.7 μm particle size; Waters) using a Vanquish Binary Pump (400μL/min flow rate; Thermo Scientific). Mobile phase A consisted of 10mM ammonium acetate and 250μL/L acetic acid in ACN:H2O (70:30, v/v). Mobile phase B consisted of IPA:ACN (90:10, v/v) with the same additives. Initially, mobile phase B was held at 2% for 2 min and then the following gradient was employed: increase to 30% over 3 min, then to 50% over 1 min, then to 85% over 14 min, and finally to 99% over 1 min where %B was held at 99% for 7 min. The column was then re-equilibrated with mobile phase B at 2% for 1.75 min before the next injection. 10μL of each extract was injected by a Vanquish Split Sampler HT autosampler (Thermo Scientific) in a randomized order. The LC system was coupled to a Q Exactive HF Orbitrap mass spectrometer (MS) through a heated electrospray ionization (HESI II) source (Thermo Scientific). Source conditions were as follows: HESI II and capillary temperature at 350C, sheath gas flow rate at 25 units, aux gas flow rate at 15 units, sweep gas flow rate at 5 units, spray voltage at |3.5 kV|, and S-lens RF at 90.0 units. The MS was operated in a polarity switching mode acquiring positive and negative full MS and MS2 spectra (Top2) within the same injection. Acquisition parameters for full MS scans in both modes were: 30,000 resolution, 1 × 10^6^ automatic gain control (AGC) target, 100 ms ion accumulation time (max IT), and 200 to 2000 m/z scan range. Data dependent (dd-MS2) scans in both modes were then collected at 30,000 resolution, 1 × 10^5^ AGC target, 50 ms max IT, 1.0 m/z isolation window, stepped normalized collision energy (NCE) at 20, 30, 40, with a 10.0 s dynamic exclusion. The resulting LC–MS/MS data were processed using Compound Discoverer 2.1 (Thermo Scientific) and LipiDex (PMID: 29705063), an in-house-developed software suite. All peaks with a 0.2 min to 23 min retention time and 100 Da to 5000 Da MS1 precursor mass were aggregated into distinct chromatographic profiles (i.e., compound groups) using a 10-ppm mass and 0.5 min retention time tolerance. Profiles not reaching a minimum peak intensity of 1x106, a maximum peak-width of 0.35, a signal-to-noise (S/N) ratio of 3, and a 3-fold intensity increase over blanks were excluded from further processing. MS/MS spectra were searched against an in-silico generated lipid spectral library containing 35,000 unique molecular compositions representing 48 distinct lipid classes. Spectral matches with a dot product score greater than 500 and a reverse dot product score greater than 700 were retained for further analysis. Lipid MS/MS spectra which contained no significant interference (<75%) from co-eluting isobaric lipids, eluted within a 3.5median absolute retention time deviation (M.A.D. RT) of each other, and found within at least 2 processed files were then identified at the individual fatty acid substituent level of structural resolution. If individual fatty acid substituents were unresolved, then identifications were made with the sum of the fatty acid substituents. Lipid quantitation was normalized to cell numbers.

### Quantification and statistics

All experiments are performed and repeated 2–5 times; data are either pooled or at least representative of 2–5 independent experiments. Data are presented as the mean ± SEM. Student’s two-tailed t-test, Mann-Whitney U test, and one-way ANOVA analyses were used to calculate the statistical significance of differences between groups, and significance was defined at p < 0.05. Statistical differences in measured variables between the experimental and control groups were assessed using Student’s t test and p < 0.05 was considered as statistically significant. Stars are p values in the following ranges: 0–0.001 = ‘***’, 0.01–0.05 = ‘**’, 0.05–0.1 = ‘*’.

## Supporting information

S1 Fig Carbomer-based adjuvant enhances DC presentation of OVA SIINFEKL peptide by H-2K^b^ MHC I molecules. (A) BMDCs were cultured in media containing OVA (1mg/ml) with or without ADJ (1%) for 6 hours. The cell surface expression of the H-2K^b^/SIINFEKL complexes was quantified by staining DCs with 25D1.16 antibodies. Plots are gated on live CD11c^+ve^ cells; Data are representative of 2 independent experiments. Error bars are SEM; *P<0.01; **P<0.001; ***P<0.0001 (One-way ANOVA).
(TIF)

S2 Fig Carbomer-based adjuvant alters antigen uptake and processing in DCs. (A) Kinetics of antigen uptake by ADJ-treated BMDCs. Cells were cultured with 20ug/ml OVA-Alexa Fluor 647 (pH insensitive dye) with or without 1% ADJ for 10 and 30 minutes. (B) Effects of ADJ on intracellular routing of antigens. BMDCs were cultured with 20ug/ml OVA labeled with the pH sensitive dye (pHAB), with or without 1% ADJ for 30 minutes. Data are representative of 4 independent experiments. Error bars are SEM; *P<0.01; **P<0.001; ***P<0.0001 (Student’s t-test).
(TIF)

S3 Fig Carbomer-based adjuvant promotes intracellular routing of OVA to early endosomes. BMDCs were pulsed with Alexa Fluor 647-OVA (60 μg/ml) and chased at the indicated time-points to assess EEA1 (A) or LAMP1 (B) co-localization. Pearson’s coefficient was calculated from 10 cells/treatment. Data are representative of 3 independent experiments Error bars are SEM; *P<0.01; **P<0.001; ***P<0.0001 (Student’s t-test).
(TIF)

S4 Fig Carbomer-based adjuvant induces intracellular H_2_O_2_ production in DCs. BMDCs were treated with 1% ADJ for 1 and 3 h with a luminogenic substrate. H_2_O_2_ levels were quantified by ROS-Glo detection solution. Data are representative of 3 independent experiments. Error bars are SEM; *P<0.01; **P<0.001; ***P<0.0001 (Student’s t-test).
(TIF)

S5 Fig Carbomer-based adjuvants do not affect proteasome activity in DCs. BMDCs were cultured with 1% ADJ for 1 or 3 h and incubated with specific luminogenic proteasome substrates Suc-LLVY (A), Z-LRR (B), and Z-nLPnLD (C) for the chymotrypsin-like, trypsin-like and caspase-like activities, respectively. Following cleavage by the proteasome, the substrate for luciferase was released and the luminescence was detected using plate reader. Data are representative of 2 independent experiments. Error bars are SEM; *P<0.01; **P<0.001; ***P<0.0001 (Student’s t-test).
(TIF)

S6 Fig Carbomer-based adjuvants reduce optical redox ratio in DCs. Optical redox ratio of unstimulated and ADJ-treated dendritic cells was calculated at the indicated time after stimulation. Representative NAD(P)H intensity (first row), FAD intensity (second row), and optical redox ratio (NAD(P)H/(NAD(P)H+FAD); third row) images of unstimulated and ADJ-treated dendritic cells. Scale bar is 10 μm. Box plots show median (central line), first and third quartiles (lower and upper hinges), the farthest data points that are no further than 1.5* the interquartile range (whiskers), and data points beyond 1.5* the interquartile range from the hinge (dots). Stars compare respective boxes to the first time point of each group (n = 22–75 cells/time point). Data are representative of 2–3 independent experiments. Error bars are SEM; *P<0.01; **P<0.001; ***P<0.0001 (Student’s t-test and one-way ANOVA).
(TIF)

S7 Fig A schematic illustration demonstrating ADJ-medicated DC cross-presentation of antigens to CD8 T cells. (TIF)
