# Supplementary figures and images for: Carbomer-based adjuvant elicits CD8 T-cell immunity by inducing a distinct metabolic state in cross-presenting dendritic cells
Source: PLoS Pathog. 2021 Jan 14;17(1):e1009168. doi: 10.1371/journal.ppat.1009168 (PMC7840022; doi:10.1371/journal.ppat.1009168)

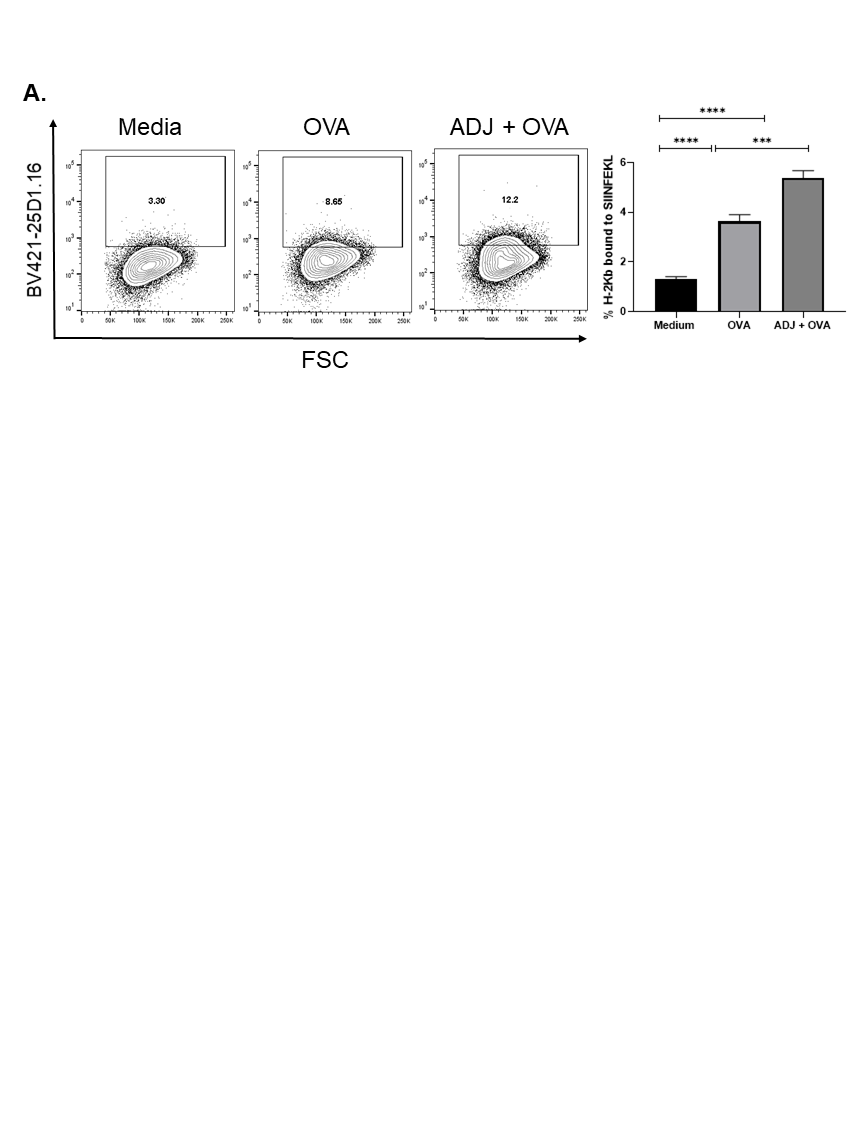

Supplement: S1 Fig — (A) BMDCs were cultured in media containing OVA (1mg/ml) with or without ADJ (1%) for 6 hours. The cell surface expression of the H-2Kb/SIINFEKL complexes was quantified by staining DCs with 25D1.16 antibodies. Plots are gated on live CD11c+ve cells; Data are representative of 2 independent experiments. Error bars are SEM; *P<0.01; **P<0.001; ***P<0.0001 (One-way ANOVA). (TIF) [file ppat.1009168.s001.TIF]

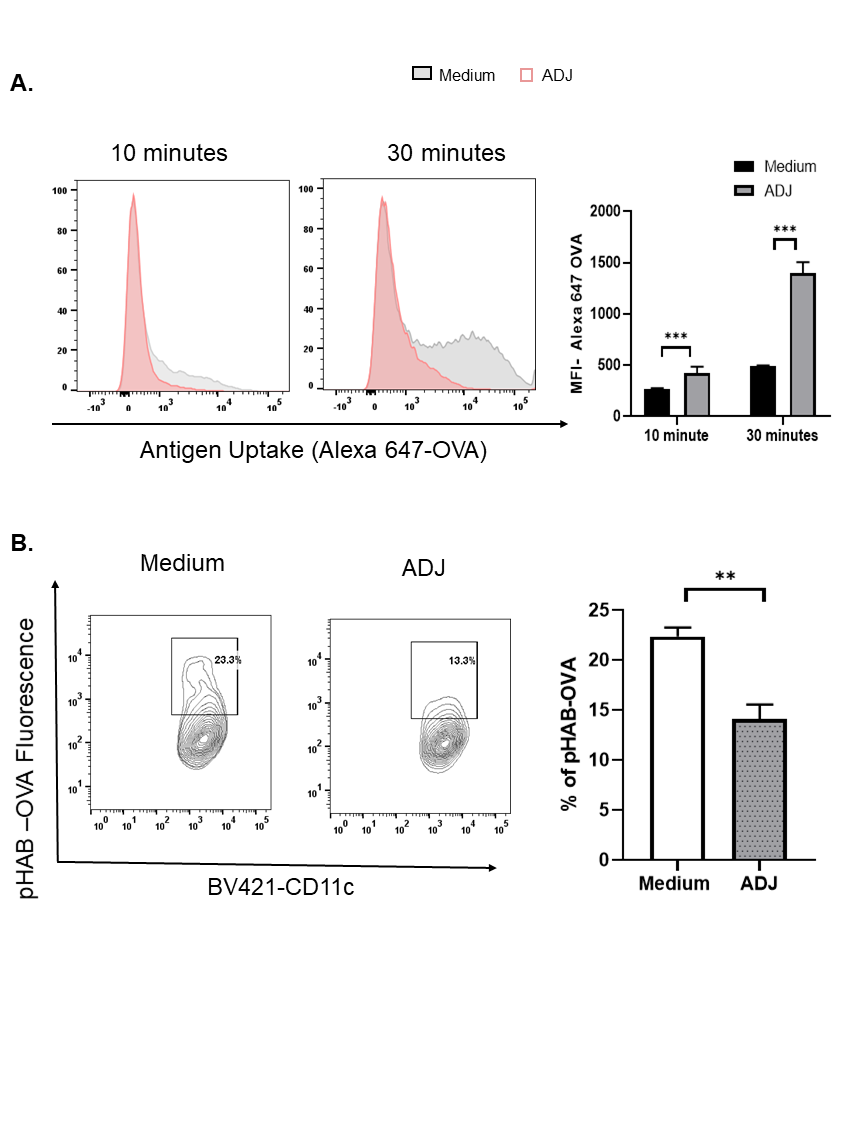

Supplement: S2 Fig — (A) Kinetics of antigen uptake by ADJ-treated BMDCs. Cells were cultured with 20ug/ml OVA-Alexa Fluor 647 (pH insensitive dye) with or without 1% ADJ for 10 and 30 minutes. (B) Effects of ADJ on intracellular routing of antigens. BMDCs were cultured with 20ug/ml OVA labeled with the pH sensitive dye (pHAB), with or without 1% ADJ for 30 minutes. Data are representative of 4 independent experiments. Error bars are SEM; *P<0.01; **P<0.001; ***P<0.0001 (Student’s t-test). (TIF) [file ppat.1009168.s002.TIF]

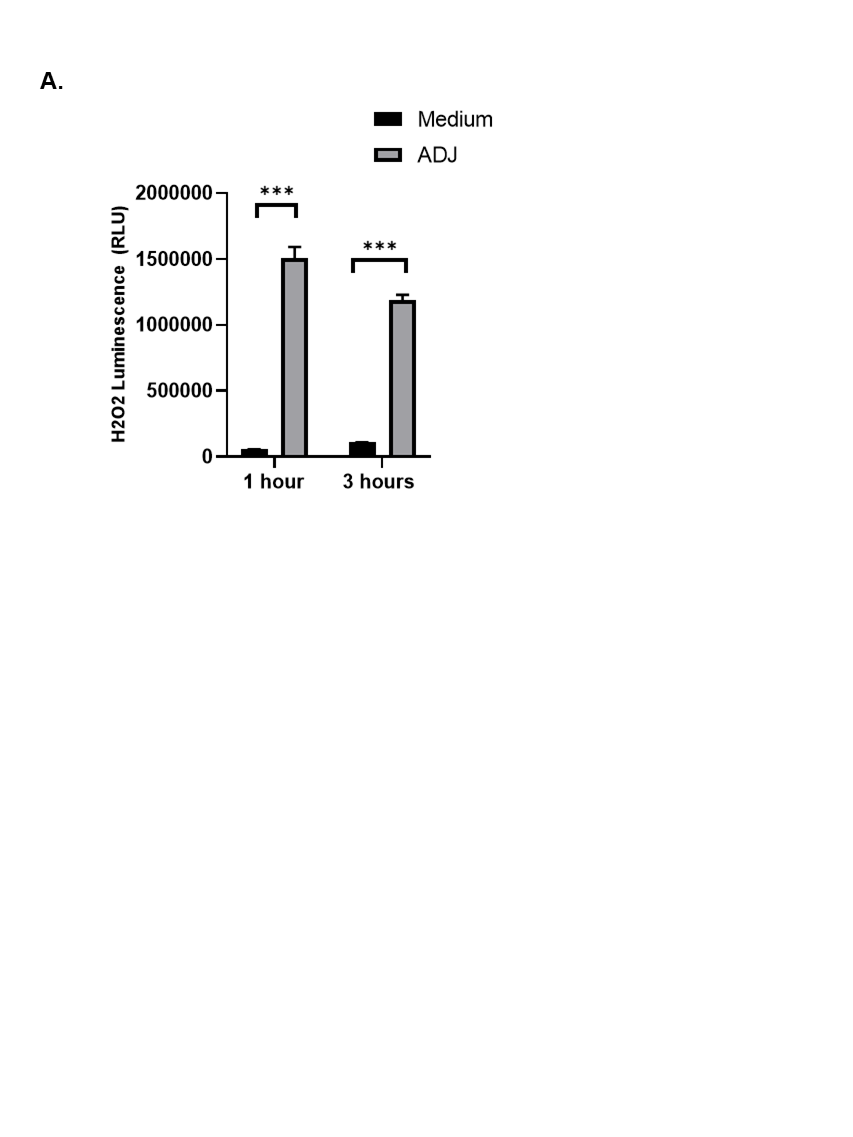

Supplement: S3 Fig — BMDCs were pulsed with Alexa Fluor 647-OVA (60 μg/ml) and chased at the indicated time-points to assess EEA1 (A) or LAMP1 (B) co-localization. Pearson’s coefficient was calculated from 10 cells/treatment. Data are representative of 3 independent experiments Error bars are SEM; *P<0.01; **P<0.001; ***P<0.0001 (Student’s t-test). (TIF) [file ppat.1009168.s003.TIF]

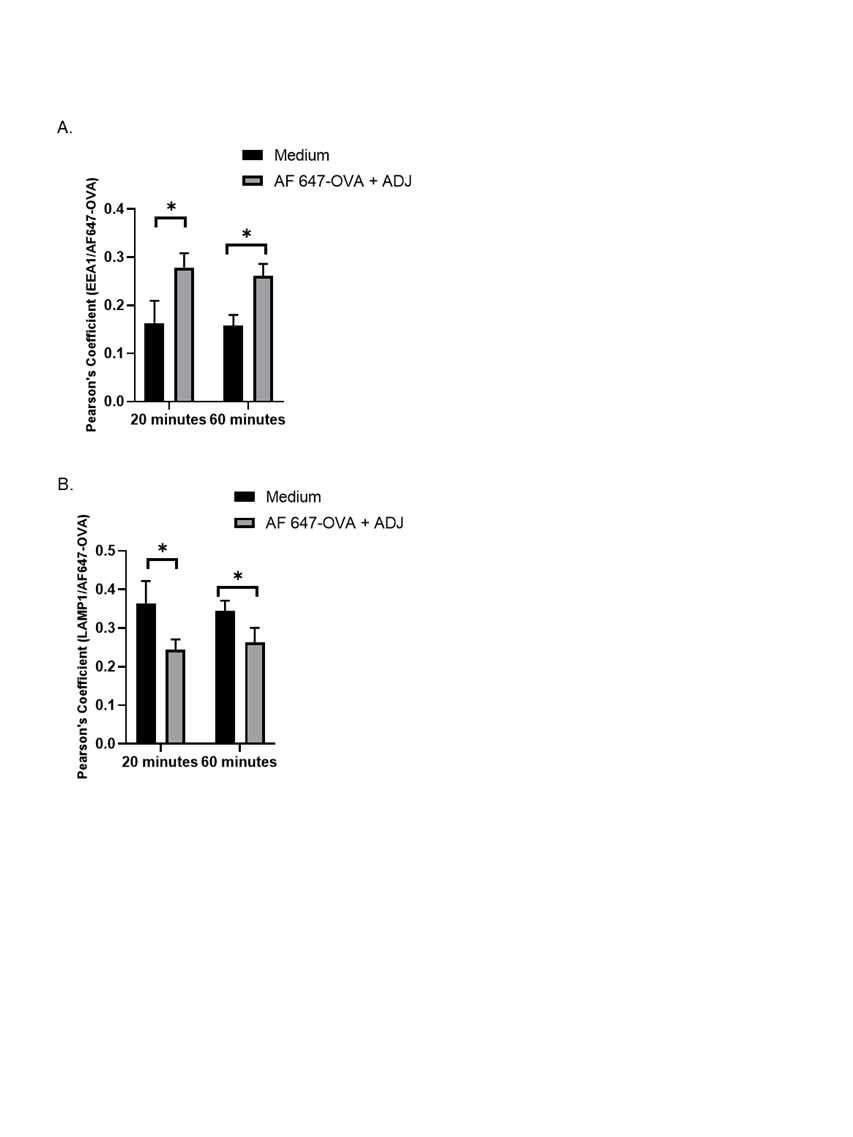

Supplement: S4 Fig — BMDCs were treated with 1% ADJ for 1 and 3 h with a luminogenic substrate. H2O2 levels were quantified by ROS-Glo detection solution. Data are representative of 3 independent experiments. Error bars are SEM; *P<0.01; **P<0.001; ***P<0.0001 (Student’s t-test). (TIF) [file ppat.1009168.s004.TIF]

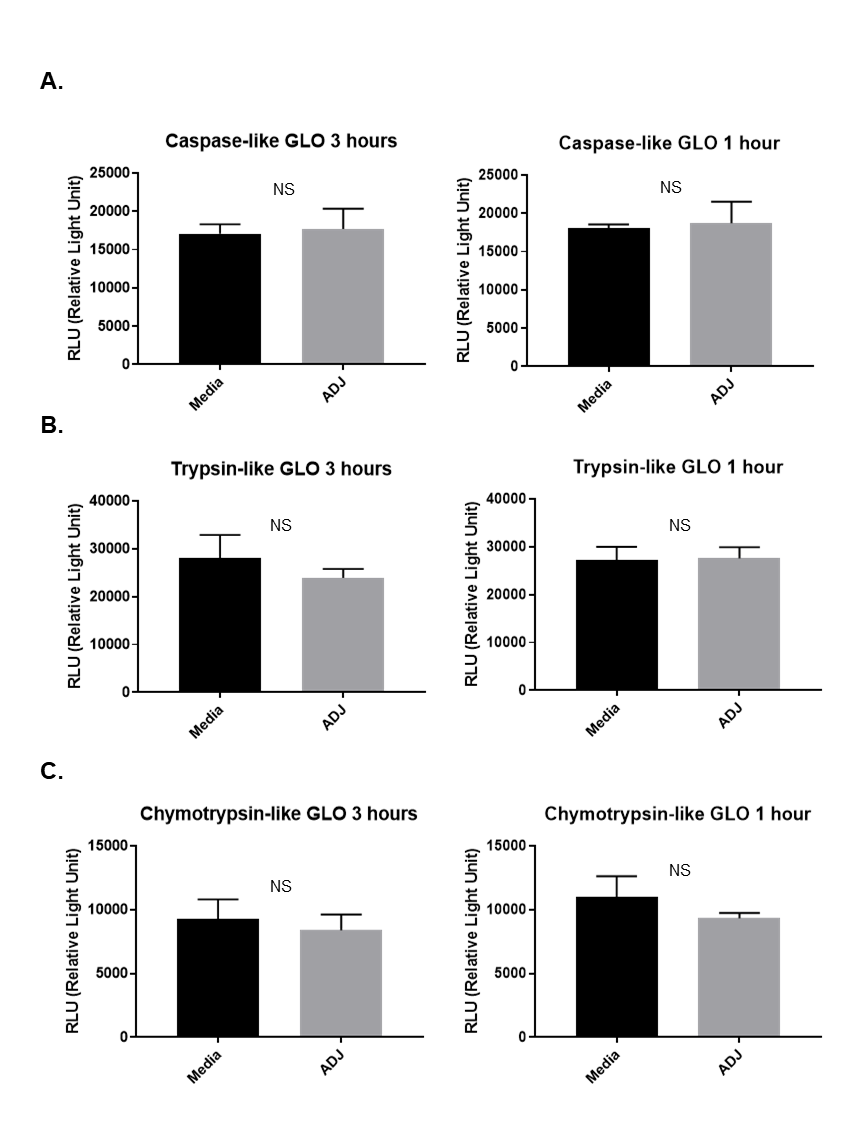

Supplement: S5 Fig — BMDCs were cultured with 1% ADJ for 1 or 3 h and incubated with specific luminogenic proteasome substrates Suc-LLVY (A), Z-LRR (B), and Z-nLPnLD (C) for the chymotrypsin-like, trypsin-like and caspase-like activities, respectively. Following cleavage by the proteasome, the substrate for luciferase was released and the luminescence was detected using plate reader. Data are representative of 2 independent experiments. Error bars are SEM; *P<0.01; **P<0.001; ***P<0.0001 (Student’s t-test). (TIF) [file ppat.1009168.s005.TIF]

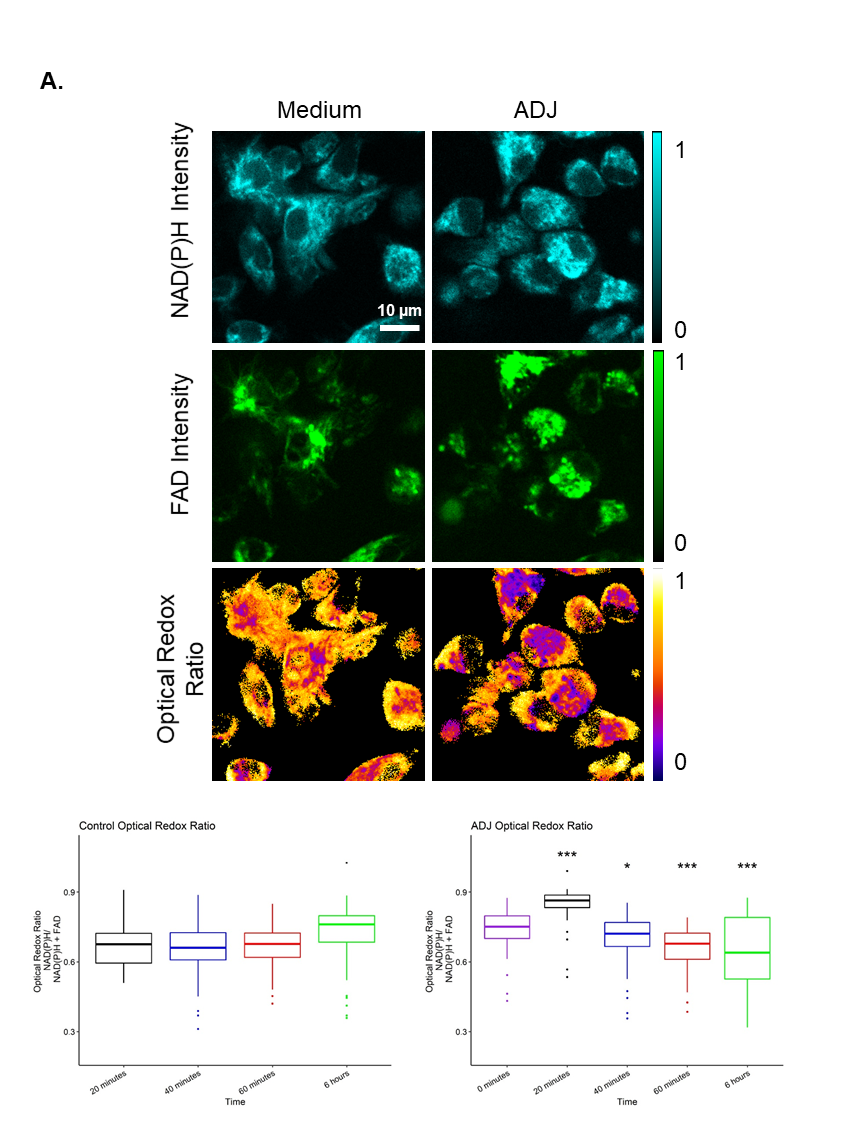

Supplement: S6 Fig — Optical redox ratio of unstimulated and ADJ-treated dendritic cells was calculated at the indicated time after stimulation. Representative NAD(P)H intensity (first row), FAD intensity (second row), and optical redox ratio (NAD(P)H/(NAD(P)H+FAD); third row) images of unstimulated and ADJ-treated dendritic cells. Scale bar is 10 μm. Box plots show median (central line), first and third quartiles (lower and upper hinges), the farthest data points that are no further than 1.5* the interquartile range (whiskers), and data points beyond 1.5* the interquartile range from the hinge (dots). Stars compare respective boxes to the first time point of each group (n = 22–75 cells/time point). Data are representative of 2–3 independent experiments. Error bars are SEM; *P<0.01; **P<0.001; ***P<0.0001 (Student’s t-test and one-way ANOVA). (TIF) [file ppat.1009168.s006.TIF]

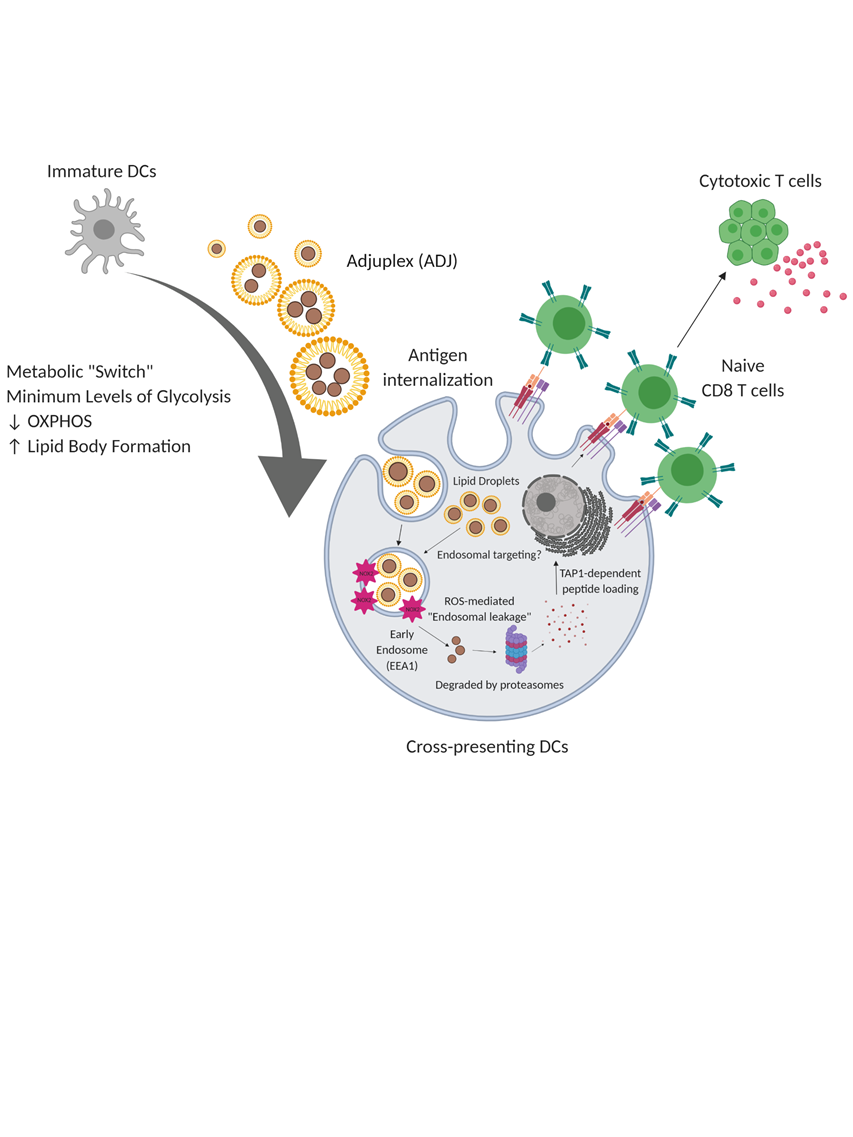

Supplement: S7 Fig — (TIF) [file ppat.1009168.s007.TIF]
